# NanoSIMS observations of mouse retinal cells reveal strict metabolic controls on nitrogen turnover

**DOI:** 10.1186/s12860-020-00339-1

**Published:** 2021-01-11

**Authors:** Elisa A. Bonnin, Eugenio F. Fornasiero, Felix Lange, Christoph W. Turck, Silvio O. Rizzoli

**Affiliations:** 1 Department of Neuro- and Sensory Physiology, University Medical Center Göttingen, Excellence Cluster Multiscale Bioimaging, 37073 Göttingen, Germany; 2 Center for Biostructural Imaging of Neurodegeneration (BIN), 37075 Göttingen, Germany; 3 Department of Nanobiophotonics, Max Planck Institute for Biophysical Chemistry, 37077 Göttingen, Germany; 4 Clinic for Neurology, University Medical Center Göttingen, 37075 Göttingen, Germany; 5 Proteomics and Biomarkers, Max Planck Institute of Psychiatry, Munich, Germany

**Keywords:** Secondary ion mass spectrometry, Imaging, Metabolic labelling, Protein turnover, Retina

## Abstract

**Background:**

Most of the cells of the mammalian retina are terminally differentiated, and do not regenerate once fully developed. This implies that these cells have strict controls over their metabolic processes, including protein turnover. We report the use of metabolic labelling procedures and secondary ion mass spectrometry imaging to examine nitrogen turnover in retinal cells, with a focus on the outer nuclear layer, inner nuclear layer, and outer plexiform layer.

**Results:**

We find that turnover can be observed in all cells imaged using NanoSIMS. However, the rate of turnover is not constant, but varies between different cellular types and cell regions. In the inner and outer nuclear layers, turnover rate is higher in the cytosol than in the nucleus of each cell. Turnover rates are also higher in the outer plexiform layer. An examination of retinal cells from mice that were isotopically labeled very early in embryonic development shows that proteins produced during this period can be found in all cells and cell regions up to 2 months after birth, even in regions of high turnover.

**Conclusions:**

Our results indicate that turnover in retinal cells is a highly regulated process, with strict metabolic controls. We also observe that turnover is several-fold higher in the synaptic layer than in cell layers. Nevertheless, embryonic proteins can still be found in this layer 2 months after birth, suggesting that stable structures persist within the synapses, which remain to be determined.

**Supplementary Information:**

The online version contains supplementary material available at 10.1186/s12860-020-00339-1.

## Background

The mammalian retina is a highly important sensory organ, as it is in the retina that the first steps of vision processing occur [1]. Photoreceptors in the mammalian retina are terminally differentiated—that is, no neurogenesis occurs after embryonic development to replace dead or damaged cells [2]. Similarly, the structures of the mammalian eye have been observed to be home to various long-lived proteins (LLPs), which are proteins that are observed to have little to no turnover compared to normal proteins [3–6]. Rates of protein turnover in retinal cells, however, are of interest to researchers because a thorough understanding of metabolic processes in mammalian cells is important to studies of retinal regeneration using stem cell treatments, an area of active study [7].

To further investigate retinal turnover, we took advantage of the high spatial resolution of nanoscale secondary ion mass spectrometry (NanoSIMS), which can have a spatial resolution of up to ~ 50 nm using the cesium positive ion source [8]. We combined these techniques with isotopic labeling procedures, where mice were fed food labeled with ^15^N for specific periods of time. These mice were divided into two groups, adult mice which were fed isotopically enriched food for a specific period of time (5 days, 14 days, 21 days, 60 days), and mice that were fed with isotopically labeled food beginning on embryonic day 7 through isotopically labeled food provided to the dam during pregnancy and after delivery until postnatal day 56. This enabled us to use ^15^N as a marker for the newly produced cellular elements [9], which in turn allows us to estimate the turnover of these structures.

The diet employed replaced the naturally occurring ^14^N with ^15^N for all molecules and metabolites. However, the ensuing sample preparation, in which retinas were fixed, ethanol-dried and embedded in a plastic resin, removed most of the lipids and small metabolites from the cells while preserving proteins and nucleic acids, which are relatively large molecules that can be well fixed. Although this method cannot characterize specific proteins, these experiments enabled an analysis of the turnover of nitrogen in these cells. Because nitrogen is a major component of many proteins and nucleic acids, areas of high nitrogen turnover suggest similarly high rates of protein turnover particularly in the nuclei, where DNA replication would substantially increase the local ^15^N levels. Conversely, in complete absence of any protein turnover, we would expect that no ^15^N would be incorporated into the cells.

In this experiment we primarily examined the photoreceptor cells within the outer nuclear layer (ONL), the synapses among and between retinal photoceptors, horizontal cells, and bipolar cells which can be found in the outer plexiform layer (OPL), and the bipolar and amacrine cells of the inner nuclear layer (INL) [10–12]. These analysis regions were chosen for their distinctive structures, which are easy to distinguish in SIMS even without the aid of correlative microscopy, and their importance to the visual function of the retina as a whole. We report in this study the first use of NanoSIMS techniques to visualize turnover within retinal cells, and also show that turnover rates vary between different cells and cell regions, although turnover rates increase in similar fashions over time.

## Results

**Fig. 1:**
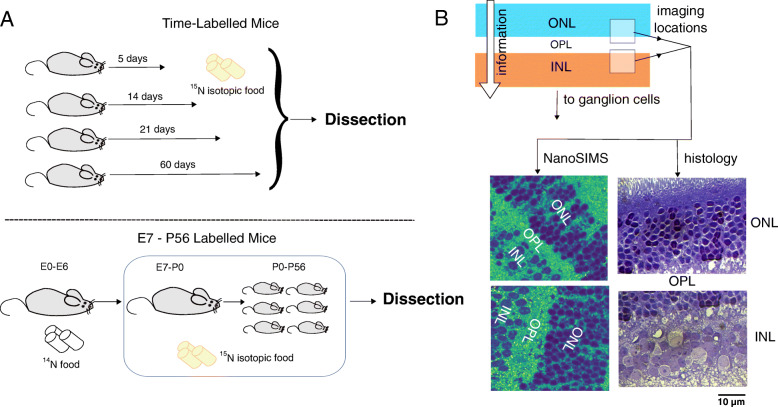
Schematic of experiment. **a** In the time-labelled treatment (top), adult mice were isotopic food for a period of 5, 14, 21, or 60 days, after which the animals were sacrificed and the retinas removed. A second group of mice (bottom) were fed ^15^N-labelled food from E7 to P56, after which animals were sacrificed and retinas removed. **b** Images were then taken from the ONL, OPL, and INL regions using NanoSIMS. Similarly, histology images were taken of the ONL and INL regions (100x). Imaging locations are represented in the schematic as semi-opaque white boxes. Scale bar for both images: 10 μm

**Fig. 2:**
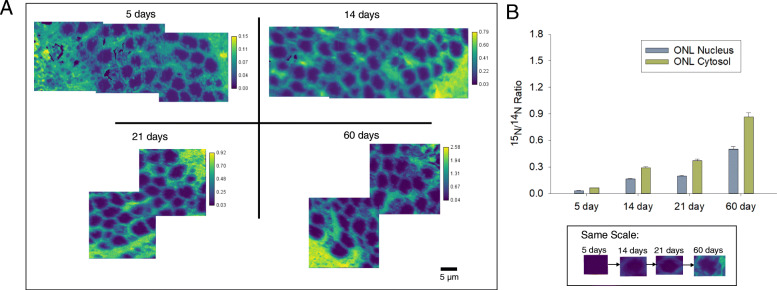
Summary of results from the outer nuclear layer (ONL) across all ^15^N-labelled treatments. **a** NanoSIMS ^15^N/^14^N cts/cts ratio images of the ONL from samples labeled with ^15^N for 5 days, 14 days, 21 days, and 60 days. All images are autoscaled to enhance contrast. Color scales are in units of ^15^N/^14^N (cts/cts). Inset: Single cells from each treatment displayed using the same color scale as the 60 day image in (**a**). **b** Average ^15^N/^14^N ratios across all treatments divided into the nucleus and cytosol areas of the ONL. Error bars represent 1 standard deviation (1σ)

**Fig. 3:**
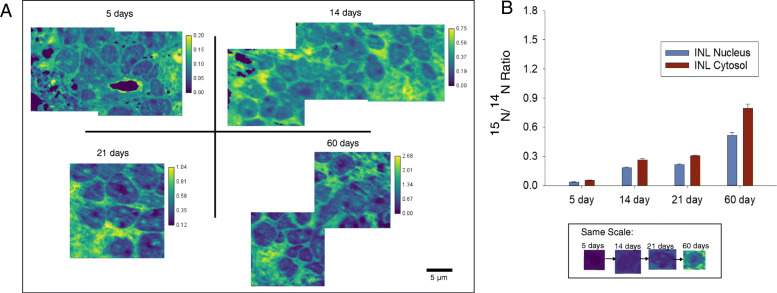
Summary of results from the inner nuclear layer (INL) across all ^15^N-labelled treatments. **a** NanoSIMS ^15^N/^14^N cts/cts ratio images of the INL from samples labeled with ^15^N for 5 days, 14 days, 21 days, and 60 days. All images are autoscaled to enhance contrast. Color scales are in units of ^15^N/^14^N (cts/cts). Inset: Single cells from each treatment displayed using the same color scale as the 60 day image in (**a**). **b** Average ^15^N/^14^N ratios across all treatments divided into the nucleus and cytosol areas of the INL. Error bars represent 1 standard deviation (1σ)

**Fig. 4:**
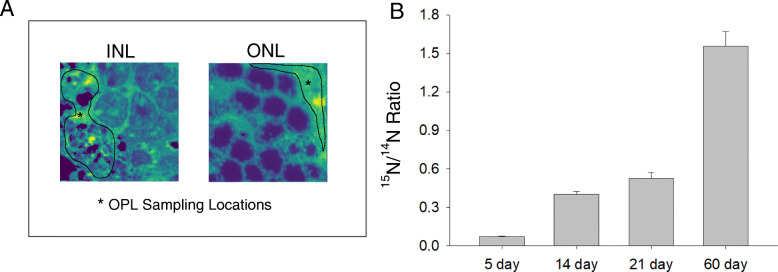
Summary of results from the outer plexiform layer (OPL) across all ^15^N-labelled treatments. **a** OPL ^15^N/^14^N ratios were obtained from both ONL and INL images. The dashed lines show typical sampling locations for the OPL. **b** Average ^15^N/^14^N ratios across all ^15^N-labelled treatments in the OPL. Error bars represent 1 standard deviation (1σ)

**Fig. 5:**
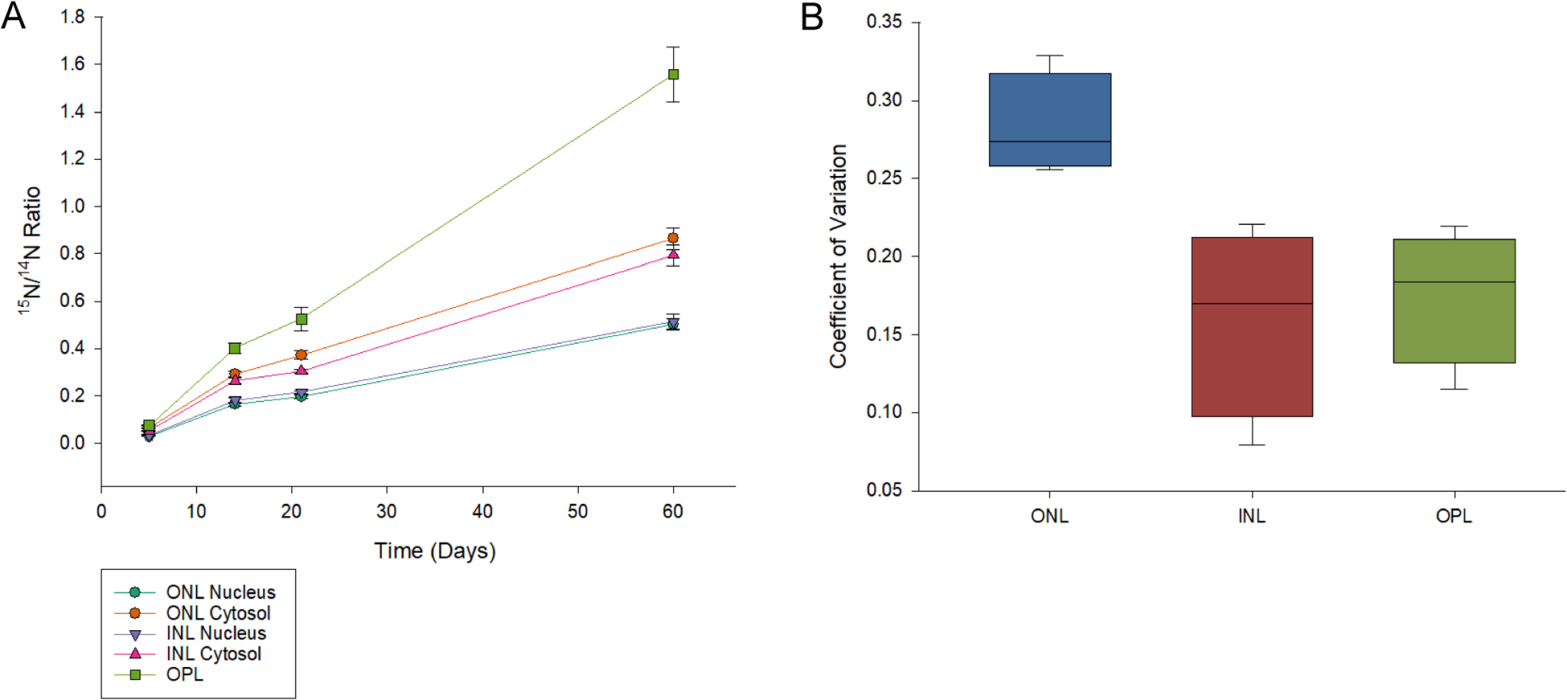
**a** Average ^15^N/^14^N ratios for each cellular region plotted against the number of experimental days in each treatment (5 days, 14 days, 21 days, and 60 days). Error bars represent 1 standard deviation (1σ). **b** Summary of coefficients of variation for the ONL (combining nucleus and cytosol), INL (combining nucleus and cytosol) and OPL regions of the timed-labelling experiments. Coefficients of variation were calculated as CV = Standard Deviation / Mean. Solid lines represent the average coefficient of variation across all four treatments. The boxed area represents the full range of coefficients of variation for all four treatments of each region. Error bars represent 1 standard deviation (1σ)

^15^N/^14^N NanoSIMS images acquired from the ONL, OPL, and INL of all timed-labelling treatments showed evidence of abundant turnover during the experimental period (Fig. 1). The rate of turnover, however, was not constant and varied between different cellular regions. In all cases, the ONL and INL nuclei had a lower apparent rate of turnover than their corresponding cytosol regions (Figs. 2 and 3). The OPL had a substantially higher turnover rate than both regions of the INL and ONL (Fig. 4). These relationships are statistically significant at the 95% confidence interval (*p* < 0.05) (Table S1). Across all treatments, the ONL and INL nuclei had statistically indistinguishable amounts of turnover (*p* > 0.05), and the ONL and INL cytosol regions were statistically indistinguishable from each other except in the case of the 5 day treatments (Table S1). However, variation in the ^15^N/^14^N ratios of the ONL is greater than in the INL. (Fig. 5, Supplemental).

This larger variation applied both to the nuclei and cytosol regions. The amount of turnover for all regions rose with time at a consistent rate across all treatments, increasing by roughly five-fold (5.1 ± 0.3 times) between 5 days and 14 days, then increasing by 1.2 ± 0.1 times between 14 and 21 days, and finally increasing by 2.6 ± 0.3 times between 21 and 60 days. This observed rise in ^15^N/^14^N is statistically significant at the 95% level of confidence (*p* < 0.05) for all treatments and regions (Table S2).

These data can be used to calculate an apparent protein half-life in each of the retinal regions, using the formula by Schwanhäusser et al., (2011) [13] as modified in Mathieson et al., (2018) [14]. When this calculation was performed, the following apparent half-lives were observed: 96.89 ± 0.26 days for the ONL nucleus, 61.66 ± 0.11 days for the ONL cytosol, 93.72 ± 0.31 days for the INL nucleus, 66.84 ± 0.13 days for the INL cytosol and 42.02 ± 0.08 days for the OPL.

**Fig. 6:**
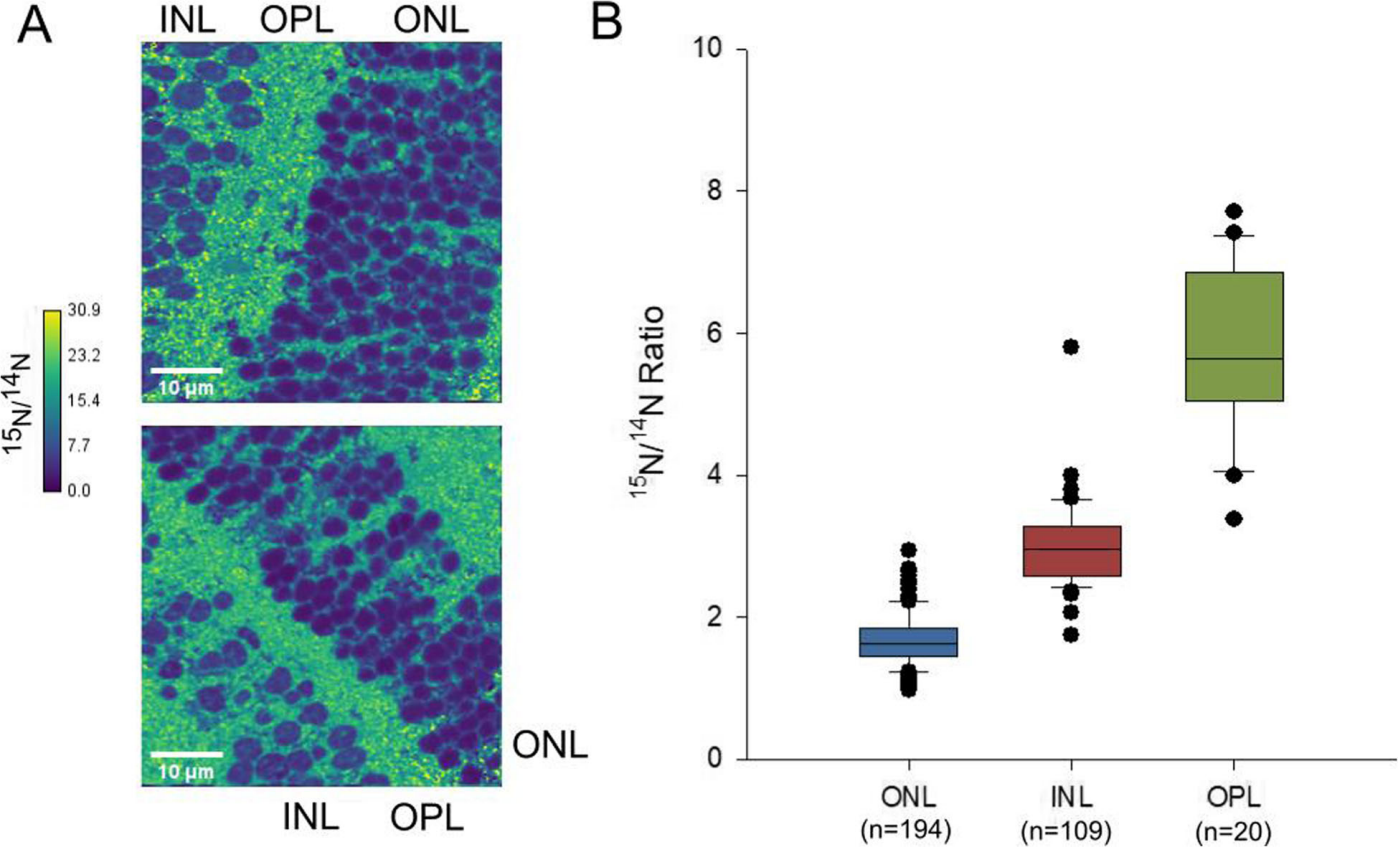
Summary of results from the outer nuclear layer (ONL), inner nuclear layer (INL), and outer plexiform layer (OPL) of mice labeled with ^15^N from E7 to P56. **a** Characteristic NanoSIMS ^15^N/^14^N (cts/cts) images of the ONL, INL, and OPL of P56 mice. Color scales are in units of ^15^N/^14^N (cts/cts). **b** ^15^N/^14^N ratios from all three regions from the P56 mice retina. Error bars represent 1 standard deviation (1σ). Outliers are depicted as black dots

Observations from mice fed entirely with ^15^N-labelled food from the seventh day of embryonic development (E7) to postnatal day 56 (P56), hereafter referred to as the ‘P56 mice’, indicated that a significant portion of the ONL and INL cells were produced prior to E7 (Fig. 6), as they still contained high levels of ^14^N which was only available to the animals during the first days of embryonic development. In these mice, the ONL (^15^N/^14^N = 1.45 ± 0.30) had a significantly lower ^15^N/^14^N ratio than the INL (^15^N/^14^N = 1.73 ± 0.61), which in turn had a significantly lower ^15^N/^14^N ratio than the OPL (^15^N/^14^N = 5.65 ± 1.59) (Table S3).

By using a simple mass model, we can compare the isotope ratios obtained from these mice with the isotope ratio we would expect if there were no embryonic proteins present. The mouse model used (male CD-1 mice, Harlan Winkelmann, Borchen, Germany), has an average weight at P56 (8 weeks after birth) of ~ 35 g, according to the information sheet provided by the supplier. For the mass of the mouse embryo at E7, we assume 0.026 g, the estimated mass at E10.5 according to Mu et al., (2008) [15]. We acknowledge that the true mass at E7 is most likely less than the mass of the embryo at E10.5, however, the difference between the mass at E10.5 and the average adult weight of 35 g is sufficiently large that the comparatively small difference in mass between E7 and E10.5 is unlikely to significantly change the results of the model. However, it is worth pointing out this uncertainty as it is possible that our expected ^15^N/^14^N ratio based on the animal’s diet is likely an underestimate.

The isotopically enriched food provided to the animals between E7 and P56 is the ^15^N-SILAM diet from Silantes (Silantes, Germany; cat. Num. 231,304,650), which has an isotopic enrichment of > 98%. Assuming then a ^14^N concentration in the food of 2%, and assuming the beginning and ending weights of the animals as provided in the previous paragraph, we estimate that the ^15^N/^14^N ratio of the animal’s organic material if no embryonic proteins remained should be ~ 49 (the isotopic ratio of the food). If all embryonic proteins remained, and they were evenly distributed across the biomass of the animal, we would expect the animal’s ^15^N/^14^N ratio to be ~ 4.4 based on its mass. This represents a point at which 99.93% of the animal is isotopically enriched. Because this number is an underestimate, the actual level of enrichment is likely to be higher than our calculations.

Our observed ^15^N/^14^N ratios from the ONL and INL are 1.45 ± 0.30 and 1.73 ± 0.61 respectively. This is lower than both the expected isotope ratio of 49 if the animal’s biomass exactly matched the isotope ratio of the diet, and the calculated ratio of 4.4 if all embryonic proteins were evenly distributed across the animal’s body and suggests a relatively high concentration of embryonic proteins in these regions. This is not unexpected, as a significant portion of the nitrogen signal in the nucleus regions is made up of DNA, which does not undergo turnover in non-dividing cells. These ratios, however, are still higher than natural abundance, suggesting that the ONL and INL did take in isotopically labeled material during development.

The OPL ^15^N/^14^N ratio (5.65 ± 1.59) is still lower than the predicted ratio based on the animal’s diet if all embryonic proteins were replaced, suggesting that the OPL layer contained substantial levels of ^14^N-containing structures (most likely proteins) at P56, almost 2 months after birth. This implies that such proteins are extremely long lived, even in a compartment with as high a turnover rate as the synaptic areas from the OPL.

## Discussion

This study reports the first observations of turnover in retinal cells using SIMS analysis and isotope labelling techniques. Our observations using mice that had been labelled with ^15^N for specific periods of time show that abundant turnover can be observed in all cells. The observation that both the ONL and INL nuclei had longer apparent protein half-lives than their corresponding cytosols and thus, slower turnover rates, is consistent with the long-standing idea that DNA, which contains a large amount of nitrogen atoms, is not replaced in retinal neurons through cell proliferation after the completion of retinal development [16–18].

However, the similar apparent half-lives of the ONL and INL are surprising, as these cells fulfill different functions and rely on different molecular mechanisms [19]. The observation that the ^15^N/^14^N ratios of the ONL were more variable than those in the INL is also surprising, as the cells of the ONL are presumably more homogenous than cells of the INL. This may indicate some level of Müller glia proliferation in the ONL, such as that which has been observed to occur after injury [20]. This would increase the rate of turnover in localized areas of the nuclear layer, however, the mechanism for this proliferation remains to be determined.

When the data are viewed separated by time (Fig. 5), we can see that although the amount of turnover varied between cell types, the rate at which this turnover increased with time was constant across all cellular structures, suggesting a tight control on cellular metabolism across all cell types. The substantially shorter apparent protein half-life of the OPL suggests that the synaptic layers are particularly unstable in metabolic terms, in agreement with the rich literature on synaptic metabolism and turnover which indicates that the synaptic proteins and organelles typically have lifetimes of only a few days in the brain [21, 22].

Data from the P56 mice, which had been labeled with ^15^N since the seventh day of embryonic development, confirm that both the ONL and INL cells are metabolically “old”, retaining proteins from the first 7 days of embryonic development close to 2 months after birth. This is consistent with previous research that shows that these cells are terminally differentiated and do not regenerate, despite the fact that portions of the cell may experience turnover and exchange over the course of the organism’s life. The OPL, despite having a significantly shorter apparent protein half-life than the other regions, also appears to contain embryonic proteins 56 days after birth, implying the existence of stable structures within the synaptic layer. The identity of these structures remains to be determined. One hypothesis is that they may represent extremely long-lived proteins that make up the extracellular matrix of neuronal and synaptic structures [21, 23].

Even in the P56 mice, there were no structures which had a ^15^N/^14^N ratio at natural abundance (~ 0.0037). All structures in the P56 specimens show some level of ^15^N incorporation into the cells, although the cells of the INL and ONL have a markedly lower ^15^N/^14^N ratio than cellular areas with higher turnover. ^15^N incorporation into these cells during this time period is not unexpected, as cell differentiation in the mouse retina is not completed until P6 (center) or P11 (periphery) [18, 24]. Retinal neurogenesis continues until roughly P7 [25], well within the period where the mice were being fed isotopically labeled food.

We also observed in these mice that ^15^N is lower in the nuclei of the ONL cells than the INL cells. This suggests that the cells of the ONL are ‘older’ than those of the INL, containing more embryonic proteins. The observation is consistent with the timing of neurogenesis in the mouse retina, as the photoreceptor cells located in the ONL form earlier in development than most of the horizontal cells in the INL [24]. Thus, it is feasible that the cells of the INL contain more of the isotopic label than ONL cells from the same time period. The observation that the amount of ^15^N incorporated into cells in the ONL varies from cell to cell is also consistent with established patterns of cellular differentiation and development, as rods and cones differentiate on separate timelines, with cones both initiating and completing their differentiation much later than rod cells. Rod nuclei make up about 97% of all photoreceptor cells in the mouse [26], and the formation of rod cells occurs between E13-P5, thus, the amount of new, ^15^N-labelled material into the photoreceptors may exhibit variation.

An interesting finding from both the P56 and the timed-labelling datasets is that in the timed-labelling experiment, there was very little difference between the observed rate of turnover in the INL compared to the ONL. However, in the P56 mice, the INL had a higher ^15^N/^14^N than the ONL, suggesting stronger ^15^N incorporation and thus, higher turnover. This further indicates that the difference in turnover rates between the ONL and INL in the P56 mice are due to differences in the growth and differentiation of these cells during development. Once these cells are mature, nitrogen turnover then occurs at a constant rate. This implies that the ONL and INL cells may be maintained by similar processes and mechanisms after initial differentiation, which remain to be determined.

Imaging with NanoSIMS is a highly time-intensive technique that limits the number of samples that can be measured for each experiment. Similarly, the isotope labelling methods used to create both the timed-labelling mice and the P56 mice are expensive treatments, which reduce the number of available samples. Thus, we acknowledge that our results, which examine retinal cells from one mouse per treatment for the timed-labelling experiments and which pool results from two mice for the P56 experiments are limited by sample size. However, these results show that the cells of the various retinal layers are complex structures, with varying mechanisms for turnover and change.

Additionally, these results serve as proof-of-principle for further, more targeted approaches with NanoSIMS, and highlight the usefulness of NanoSIMS techniques for examining subecellular metabolic processes in cells. While this study represents the first use of NanoSIMS to examine nitrogen turnover in the retina, the use of isotope labelling in conjunction with the high-resolution imaging techniques associated with SIMS is an established method for obtaining subcellular information across spatial and temporal scales [27]. The use of isotope labelling to examine turnover and the growth of new cells has previously been performed in cardiomyocytes [28]. Similarly, the application of stable isotope labels to highlight cells that were formed in early development, such as our P56 cells, has also been performed in a previous study which examined long-lived proteins in the mouse liver, pancreas, and brain [29]. It is likely that the contribution of NanoSIMS techniques to this field will increase in future years as novel and creative applications of NanoSIMS imaging and stable isotope labelling are developed.

However, one enduring limitation of NanoSIMS imaging is that while chemical information can be obtained at high resolution, matching this chemical information with corresponding structures is difficult without employing some form of correlative microscopy (for example, see: [30–32]). In the case of this study, we took simple histology images of the cellular areas being measured (Fig. 1 and Supplemental). From these images, we can see that the cellular structures observed between the NanoSIMS and the optical microscope are similar.

Despite this, it can be difficult to state with certainty which chemical structures are contributing most strongly to observed turnover. For example, microRNA, which also contains high levels of nitrogen, has been observed to have high turnover in retinal neurons [33]. Similarly, protein synthesis in the retina has been observed to be affected by light exposure [34, 35]. The extent to which light exposure may affect the observed turnover rates has not been evaluated in this study. The contribution of this turnover to the observed incorporation of ^15^N into retinal cells is yet to be determined and future research will be needed to better constrain these processes and effects.

## Conclusions

Nitrogen turnover in the ONL, OPL, and INL regions of the retina is highly regulated, and increases at a consistent rate over time across all cells and cell regions. Despite the INL and ONL cells having highly different structures and functions, there is no discernable difference in turnover rates between these cells after initial differentiation, which may indicate that these cells are maintained by similar processes.

Turnover in the OPL is several-fold higher than within the cells. However, despite this higher turnover, the OPL contains embryonic proteins even 2 months after birth, suggesting that the synaptic layer contains stable structures with low turnover and long lifetimes. The identity of these structures is currently unknown, but could be the subject of future research.

These results represent the first examination of turnover in retinal cells using NanoSIMS imaging techniques and highlight the potential of NanoSIMS analysis techniques and isotope labelling for further examinations of turnover and cellular age in organisms. Further applications of this technique to other sensory organs or cellular regions are likely to be forthcoming.

## Methods

### Acquisition and preparation of retinal samples

All timed-labelling mouse experiments were approved by the local authority, the Lower Saxony State Office for Consumer Protection and Food Safety (Niedersächsisches Landesamt für Verbraucherschutz und Lebensmittelsicherheit). Adult (> 3.5 months old) wild-type male mice (C57BL/6JRj) were purchased from Janvier Labs (Germany). Mice were habituated to unlabeled ^14^N-SILAM diet for 1 week before labelling (Silantes, Germany; cat. Num. 231,004,650). Mice were then labeled with the ^15^N-SILAM diet (Silantes, Germany; cat. Num. 231,304,650) as described in previous studies [21, 36], for defined times (respectively 5, 14, 21 and 60 days before perfusion). The experiment was designed to sacrifice and process all animals at the same time, to avoid variability during sample processing. Animals were euthanized with CO_2_ and immediately processed for intracardial perfusion, with minor adaptations from the method previously described in Gage et al., (2012) [37]. Briefly, the heart was first exposed, after which the animal blood was washed first with cold (4 °C) phosphate-buffered saline (PBS) and then with perfusion buffer (4% PFA in PBS at pH 7.4) for 3 min at a flow rate of ~ 10 ml/min. Upon perfusion, organs were harvested, including eyes. Eyes were extracted under a stereomicroscope and post-fixed overnight in fixative at 4 °C prior to embedding.

After fixation, the extracted tissue pieces were washed three times with 0.1 M cacodylic buffer (pH 7.4, Sodium cacodylate buffer 0.2 M, 11650, Electron Microscopy Sciences) for 5 min each. For osmication and further processing we first placed the tissue pieces in 1% osmium tetroxide (4% osmium tetroxide, 19,140, Electron Microscopy Sciences, Hatfield, PA, USA) in 0.1 M cacodylic buffer (pH 7.4) for 1 h at room temperature. After 4 wash steps with double distilled water (5 min each), samples were placed in a solution of 1% uranyl acetate (uranyl acetate, powder, E22400, Science Services, Munich, Germany) in double distilled water (ddH_2_O) for 1 h at room temperature in the dark. Subsequently, we washed the samples with ddH_2_O 3 times for 5 min each. The samples were dehydrated in a graded series of ethanol (30, 50, 75 and 100%, 5 min each) with a final dehydration in propylene oxide for 5 min at room temperature prior to resin infiltration with a 1:1 mixture of propylene oxide and EPON (Embed-812, 14,121, Electron Microscopy Sciences) for 1 h and two fresh replacements of 100% EPON (first for 1 h, second over night at 4 °C). For the final embedding the samples were placed between two slides of ACLAR polymer film (ACLAR Fluoropolymer film, E50425, Science Services) and covered with fresh EPON resin. Resin curing occurred in an oven at 60 °C for 48 h.

Animal experiments in the case of the P56 mice were approved by the committee for the Care and Use of Laboratory Animals of the Government of Upper Bavaria, Germany and conducted according to current regulations for animal experimentation in Germany and the European Union (European Communities Council Directive 86/609/EEC). To gain a high incorporation rate of ^15^N at early developmental stages, male CD1 mice (Harlan Winkelmann, Borchen, Germany) were labeled with ^15^N in utero by providing isotopically labeled food to the pregnant dams and for 8 weeks post partum through feeding with a bacterial protein-based, ^15^N labeled diet (U-15 N-SILAM-Mouse, Silantes GmbH, Munich, Germany). Prior to that, for habituation, pregnant dams were first fed for 7 days with non-labeled bacterial protein-based diet (U-14 N-SILAM-Mouse, Silantes GmbH) that had the same composition as the ^15^N diet. On postnatal day 56, mice were sacrificed and tissues were collected after perfusion with 0.9% saline.

All P56 retinal samples were embedded as whole eyes in LR White (London Resin Company, Ltd.) prior to analysis. Before embedding, samples were dehydrated with increasing amounts of EtOH in ddH_2_O, 30% EtOH (1 × 10 min), 50% EtOH (1 × 10 min), and 70% EtOH (3 × 10 min). Dehydrated samples were then incubated in a 1:1 mixture of LR White and 70% EtOH for 1 h, followed by incubation in pure LR White for 1 h. Eye samples were then covered with plastic capsules (Beem Inc., West Chester, PA, USA) and embedded in LR White plus LR White accelerator (London Resin Company, Ltd.) for 30 min on a pre-cooled metal plate. Following this, samples were incubated at 60 °C for 90 min. Embedded samples were cut using an EM UC6 ultramicrotome (Leica Microsystems, Wetzlar, Germany), into 200 nm sections. Retinal cells were then identified from these samples and placed on silicon wafers (Siegert Wafer GmbH, Aachen) for NanoSIMS analysis.

### Image acquisition

SIMS analysis was performed using a NanoSIMS 50 L instrument (Cameca, France), and a Cs^+^ positive ion source. Secondary ions were generated using a primary current of ~ 30 nA (primary aperture D1 = 2). Prior to each measurement, an implantation of Cs^+^ ions was performed at high current (L1 = 20,000, D1 = 1) on an area larger than the raster size used for analysis until steady state conditions were achieved. For the timed-labelling experiments, images were taken at a raster size of 20x20μm. Retinas from one mouse per experimental treatment were analyzed using NanoSIMS. In the P56 mouse retinas, images were taken at a raster size of 50x50μm. Retina samples from two P56 mice were examined in this study. All images were taken at a resolution of 256 × 256 pixels, with 5000 cts/px, leading to an estimated resolution of ~ 78 nm/px in the timed-labelling samples, and ~ 200 nm/px in the P56 mice. Three images [3] were obtained with these settings at each location. The following masses were collected for each run: ^12^C^14^N (referred to as ^14^N in this report), ^12^C^15^N (referred to as ^15^N in this report), and ^31^P. ^31^P peak was used to mark the location of cellular structures. Each image shown in this manuscript is the result of a summation of all three image layers taken during analysis.

ONL and INL histology images (Fig. 1, Supplemental) were obtained using an Axio Imager M2 upright microscope (Zeiss, Germany), and a 100x ACHROPLAN water-immersion objective (Zeiss, Germany). Samples were immersed in ddH_2_O for analysis.

### Image processing and data analysis

NanoSIMS images were processed using a custom Matlab script (the Mathworks Inc., Natick, MA, USA), adapted from the analysis script used in Gagnon et al., (2012) [38]. Image layers were aligned relative to the second image taken for each element prior to summation. The ^14^N image was then used for thresholding and the identification of background noise. Areas with sufficiently low ^14^N counts to be considered background were removed from all images. The ^15^N/^14^N (timed-labelling experiments) and ^14^N/^15^N (P56 mice) ratio images were then obtained by dividing each data point in the numerator isotope with each data point in the denominator isotope. The resulting matrix was saved as a text file. Images were then generated using FIJI/ImageJ (NIH, Bethesda, MD, USA).

In both the time-labelled experiment and the P56 experiment, average isotope ratios for each cell and cellular region were generated by manually selecting ROIs with FIJI/ImageJ. ROIs were drawn around each cell and cellular region visible in the ^14^N images. All ROIs were then saved in FIJI/ImageJ and applied to the appropriate ratio image for each experiment. Mean isotope ratios for each individual cell and cell region were generated using the Measure tool of FIJI/ImageJ.

## Supplementary Information

**Additional file 1.**

## Data Availability

The datasets used and/or analysed during the current study are available from the corresponding author on reasonable request.

## Abbreviations

DNA: Deoxyribonucleic acid
E (as in E7 … etc.): Embryonic day
INL: Inner nuclear layer
LLP: Long-lived protein
NanoSIMS: Nanoscale secondary ion mass spectrometry
ONL: Outer nuclear layer
OPL: Outer plexiform layer
P (as in P56 … etc.): Postnatal day
PBS: Phosphate-buffered saline
PFA: Paraformaldehyde
RNA: Ribonucleic acid
SILAM: Stable isotope labelling in mammals
SIMS: Secondary ion mass spectrometry
