# Supplementary material for: NanoSIMS observations of mouse retinal cells reveal strict metabolic controls on nitrogen turnover
Source: BMC Mol Cell Biol. 2021 Jan 11;22:5. doi: 10.1186/s12860-020-00339-1 (PMC7798281; doi:10.1186/s12860-020-00339-1)
Supplement: Supplementary file 1 — Additional file 1. [file 12860_2020_339_MOESM1_ESM.pdf]

Supplemental Information for: *NanoSIMS observations of protein turnover in retinal cells*

Elisa A. Bonnin, Eugenio F. Fornasiero, Felix Lange, Christoph W. Turck, Silvio O. Rizzoli

Table of Contents

Statistical Tables 2
Histology Images 2
  5 days ONL 3
  5 days INL 4
  14 days ONL 5
  14 days INL 6
  21 days INL 8
  60 days ONL 10
  60 days INL 12
Histograms of Average N Ratios 13
  5 days 13
  14 days 14
  21 days 16
  60 days 18
  P56 Mice 19

Statistical Tables

Tables S1, S2, and S3 below show the results from statistical testing. All statistical tests used were two sample t-tests assuming equal variances. All three tables display the results of a one-tailed test, which tests whether the second group has a mean statistically higher than that of the first group ($H_0: \mu_2 \leq \mu_1$). The null hypothesis is considered rejected when $p < 0.05$ ($\alpha = 0.95$) and the t-statistic is negative.

TABLE S1. Results from statistical testing between different cellular regions for each treatment.

	5 day		14 day		21 day		60 day	
**Test**	**p**	**t stat**	**p**	**t stat**	**p**	**t stat**	**p**	**t stat**
ONL Nuc ≤ ONL Cyt	0.000	-18.0	0.000	-11.7	0.000	-9.6	0.000	-7.2
ONL Nuc ≤ INL Nuc	0.062[1]	-1.6	0.097[1]	-1.3	0.145[1]	-1.1	0.422[1]	-0.2
INL Nuc ≤ INL Cyt	0.000	-5.4	0.000	-5.4	0.000	-9.2	0.000	-5.3
ONL Cyt ≤ INL Cyt	0.001	3.3[2]	0.125[1]	1.2	0.045	1.7[2]	0.250[1]	0.7
ONL Nuc ≤ OPL	0.000	-14.3	0.000	-14.9	0.000	-11.5	0.000	-14.2
INL Nuc ≤ OPL	0.000	-11.1	0.000	-9.3	0.000	-9.2	0.000	-8.8

[1] the trend is not statistically significant

[2] the trend is significant in the opposite direction ($\mu_2 \leq \mu_1$).

TABLE S2. Results from statistical testing between different experimental time points for each cellular region.

	ONL Nucleus		ONL Cytosol		INL Nucleus		INL Cytosol		OPL	
**Test**	**p**	**t stat**	**p**	**t stat**	**p**	**t stat**	**p**	**t stat**	**p**	**t stat**
5 days ≤ 14 days	0.000	-19.8	0.000	-21.2	0.000	-18.3	0.000	-14.7	0.000	-13.2
14 days ≤ 21 days	0.000	-3.4	0.000	-4.1	0.004	-3.0	0.015	-2.4	0.009	-2.7
21 days ≤ 60 days	0.000	-11.3	0.000	-9.4	0.000	-8.62	0.000	-10.4	0.000	-5.8

TABLE S3. Results from statistical testing between different cellular regions in the P56 mice.

**Test**	**p**	**t stat**
ONL ≤ INL	0.000	-23.3
ONL ≤ OPL	0.000	-41.1
INL ≤ OPL	0.000	-17.3

Histology Images

All histology images were obtained using an Axio Imager M2 upright microscope (Zeiss, Germany), and a 100x ACHROPLAN water-immersion objective (Zeiss, Germany). Samples were immersed in ddH2O for analysis.

5 days ONL

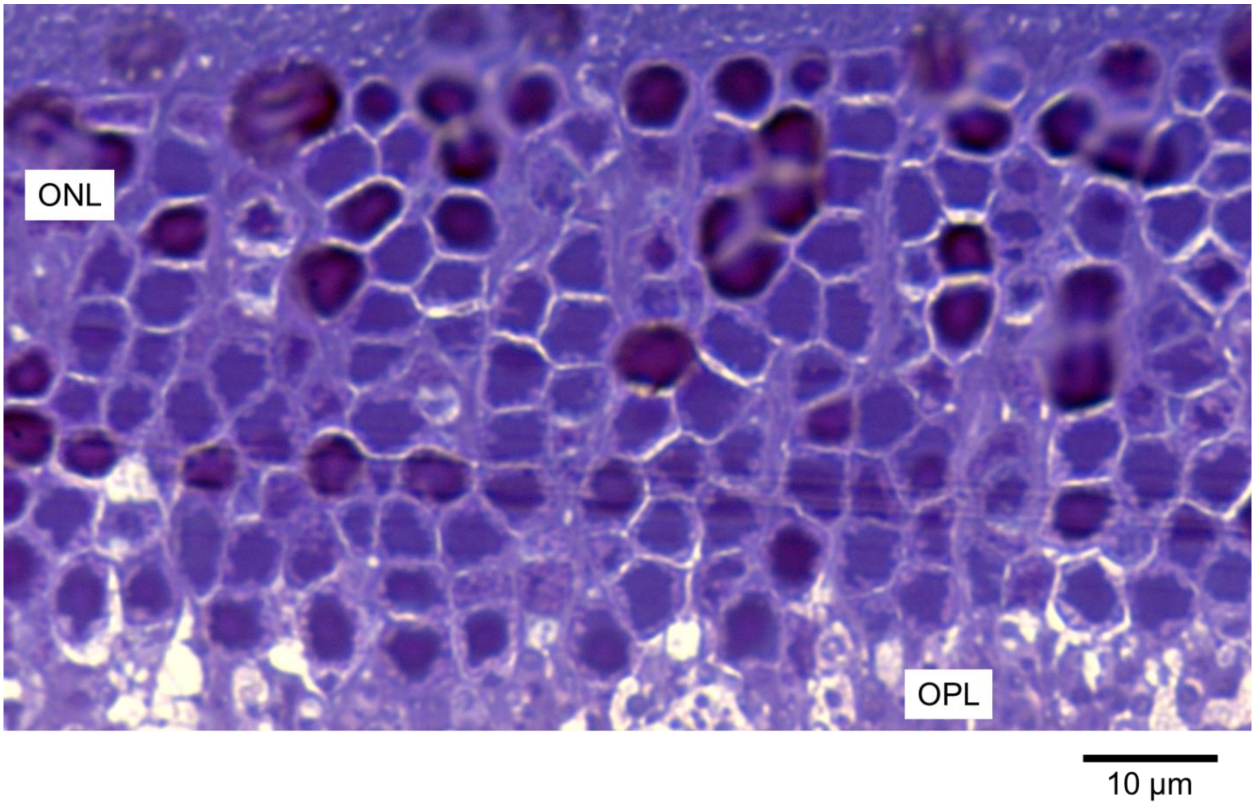

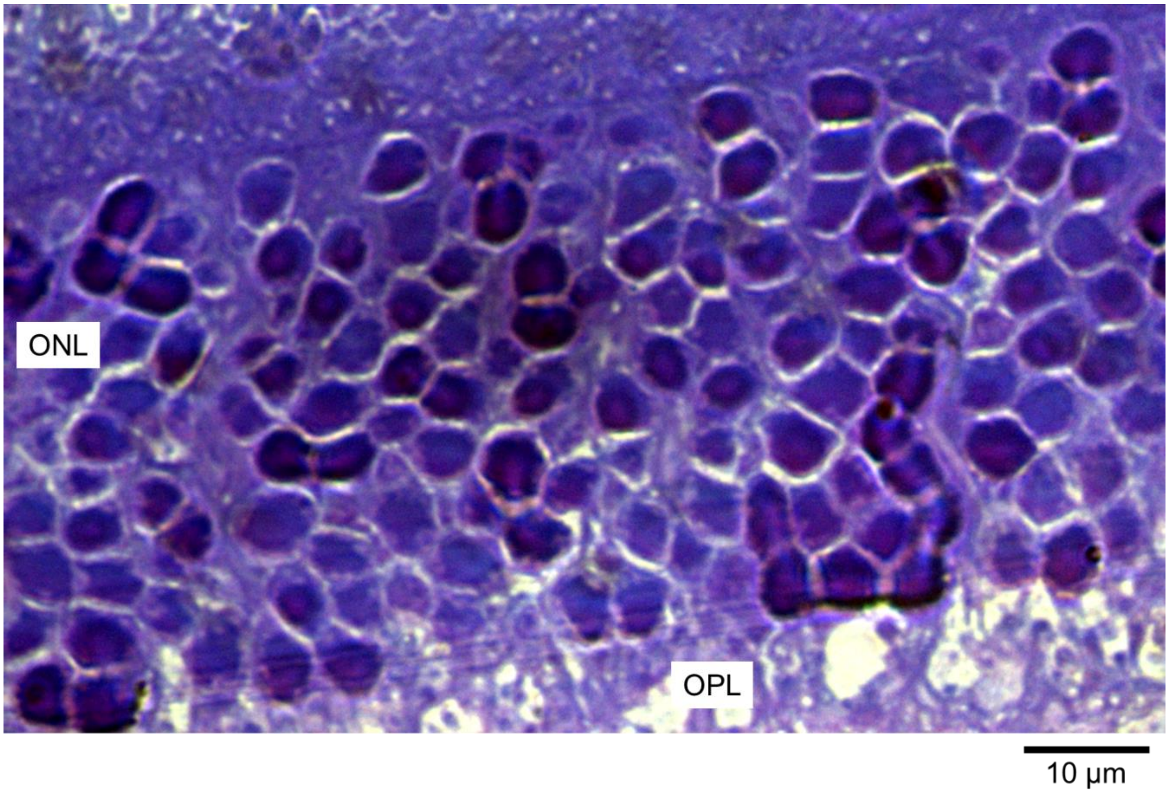

5 days INL

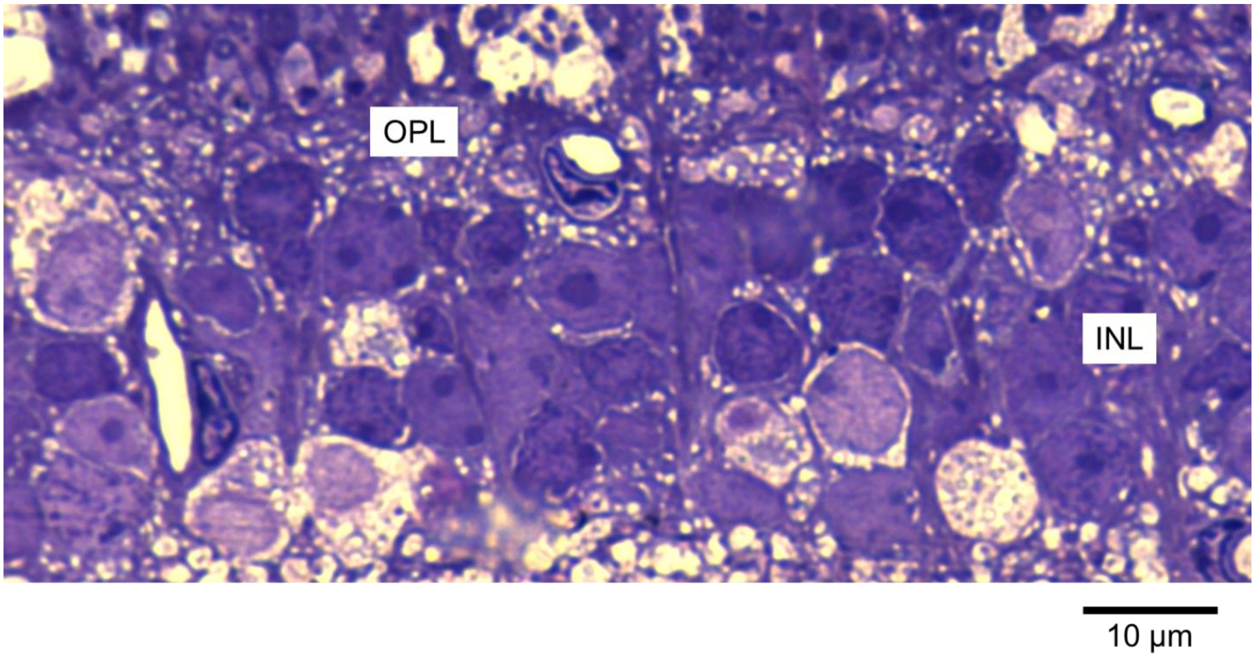

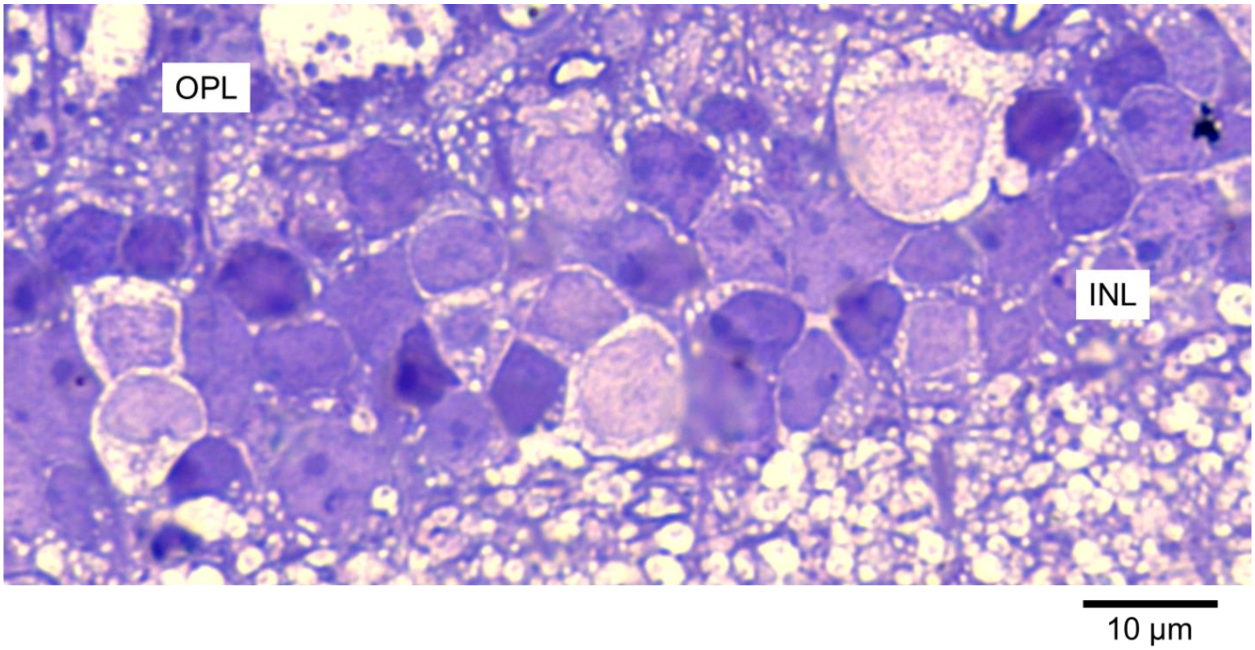

14 days ONL

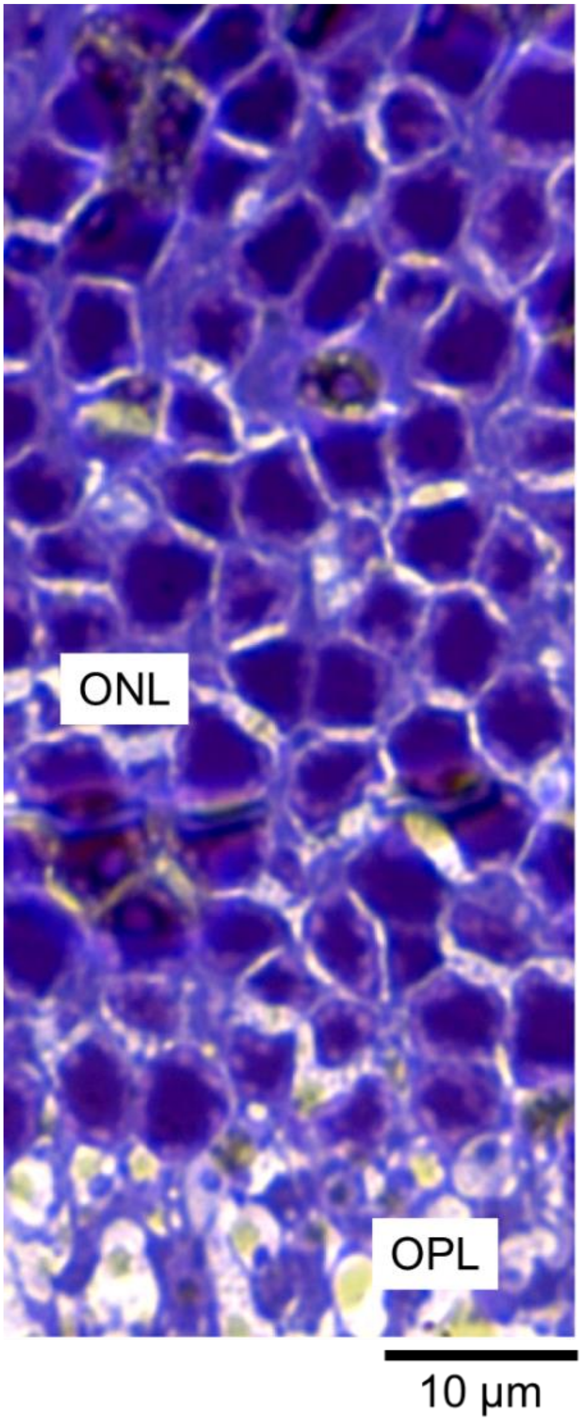

14 days INL

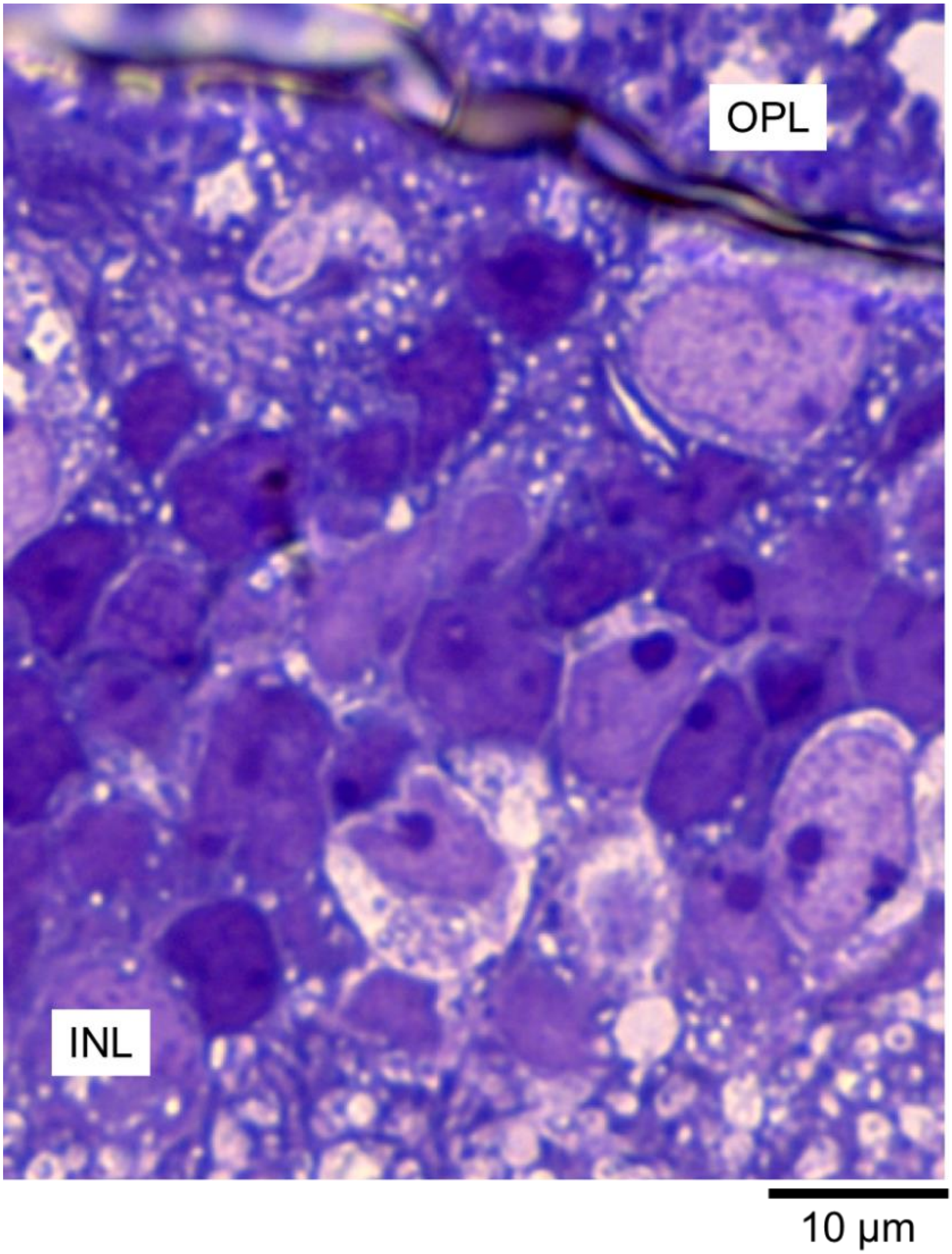

21 days ONL

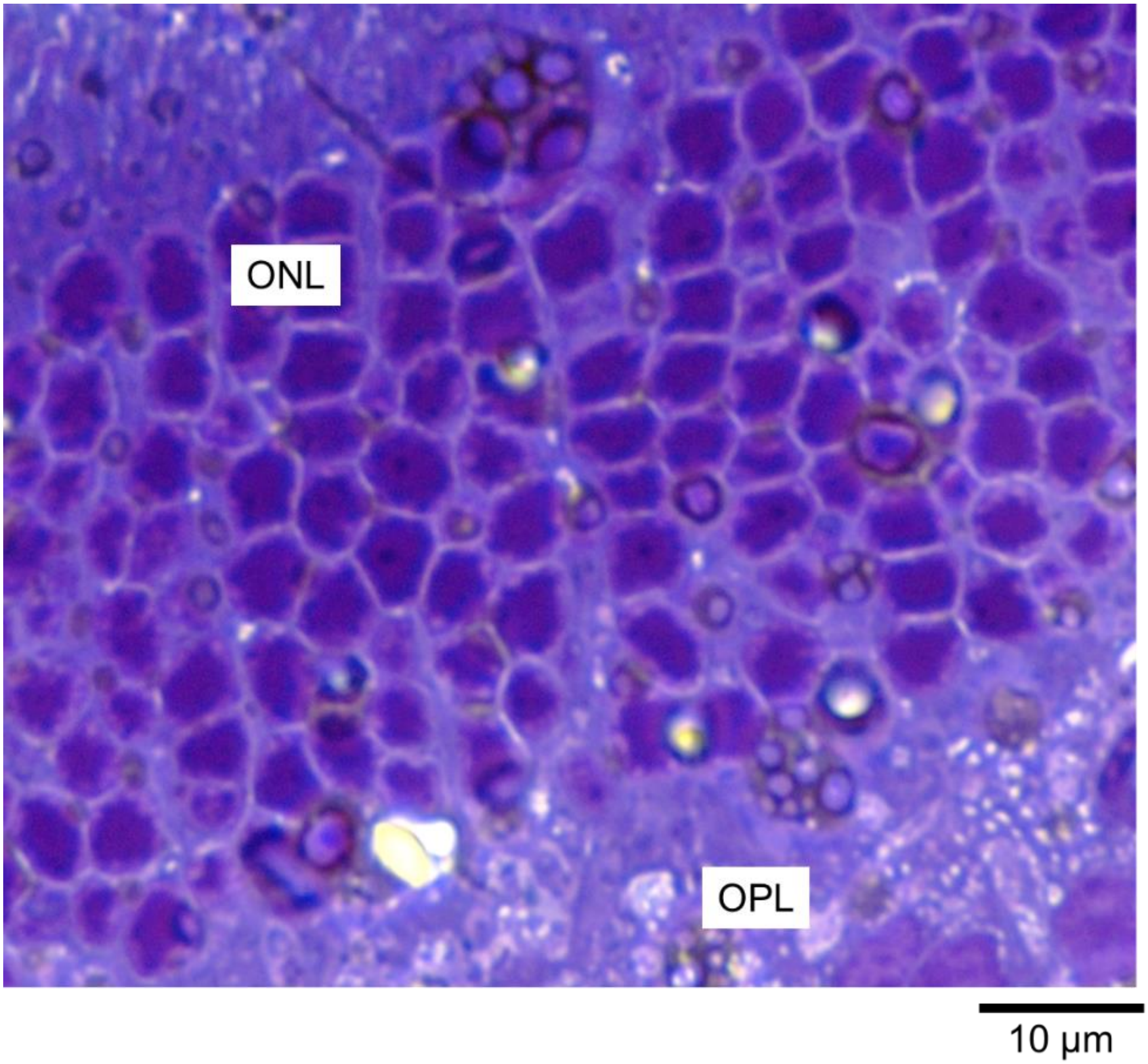

21 days INL

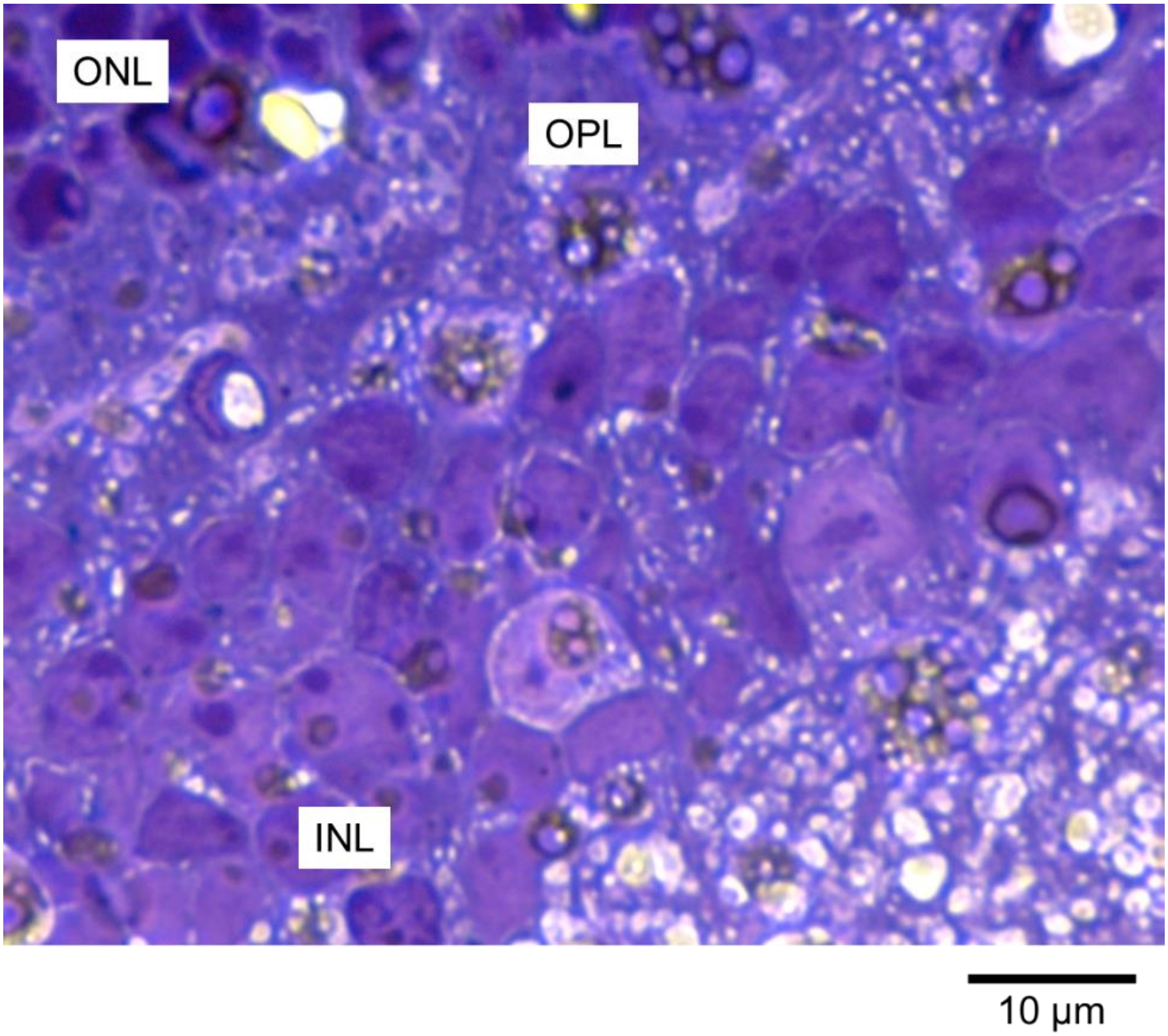

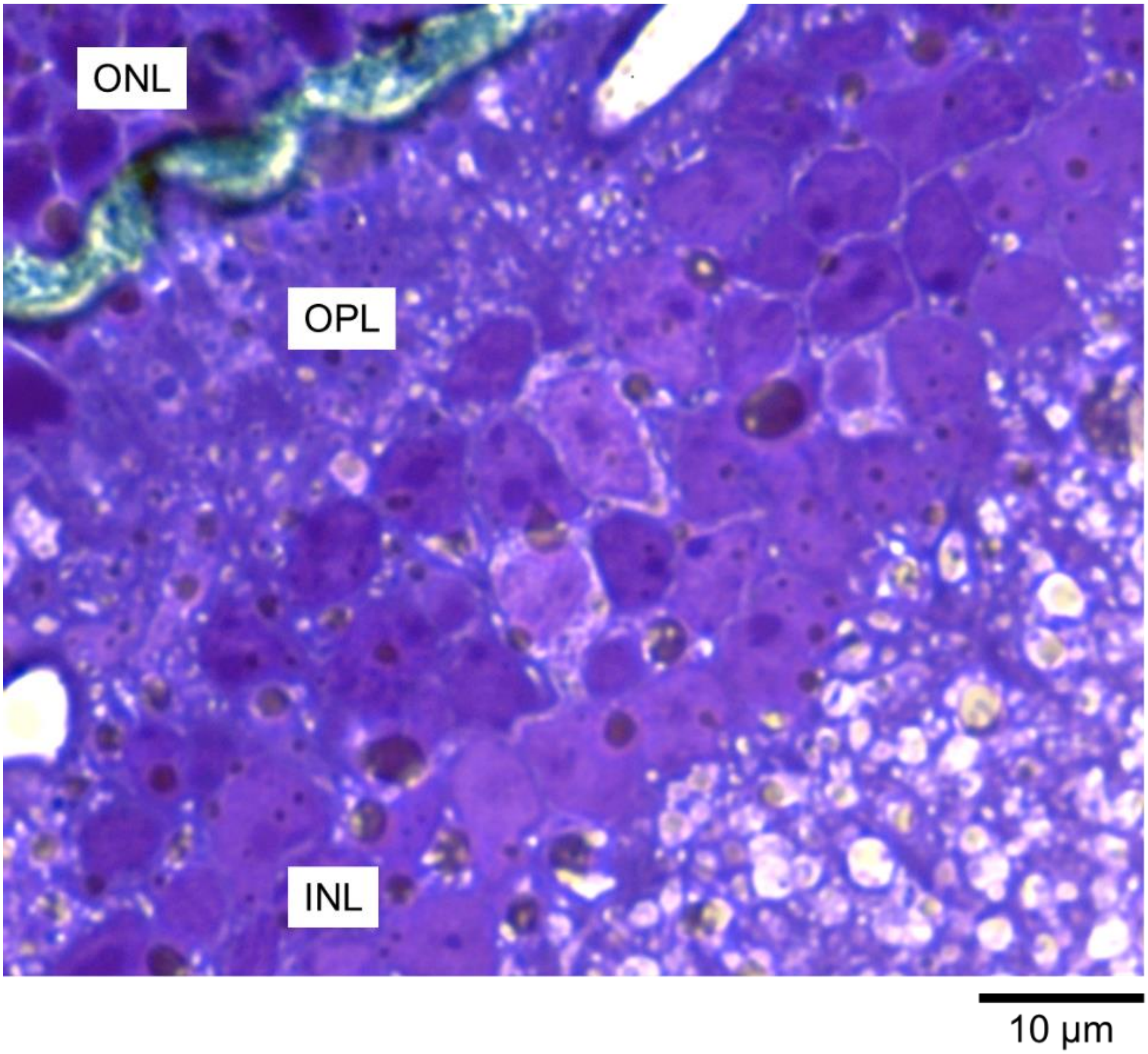
ONL
OPL
INL
10 µm

60 days ONL

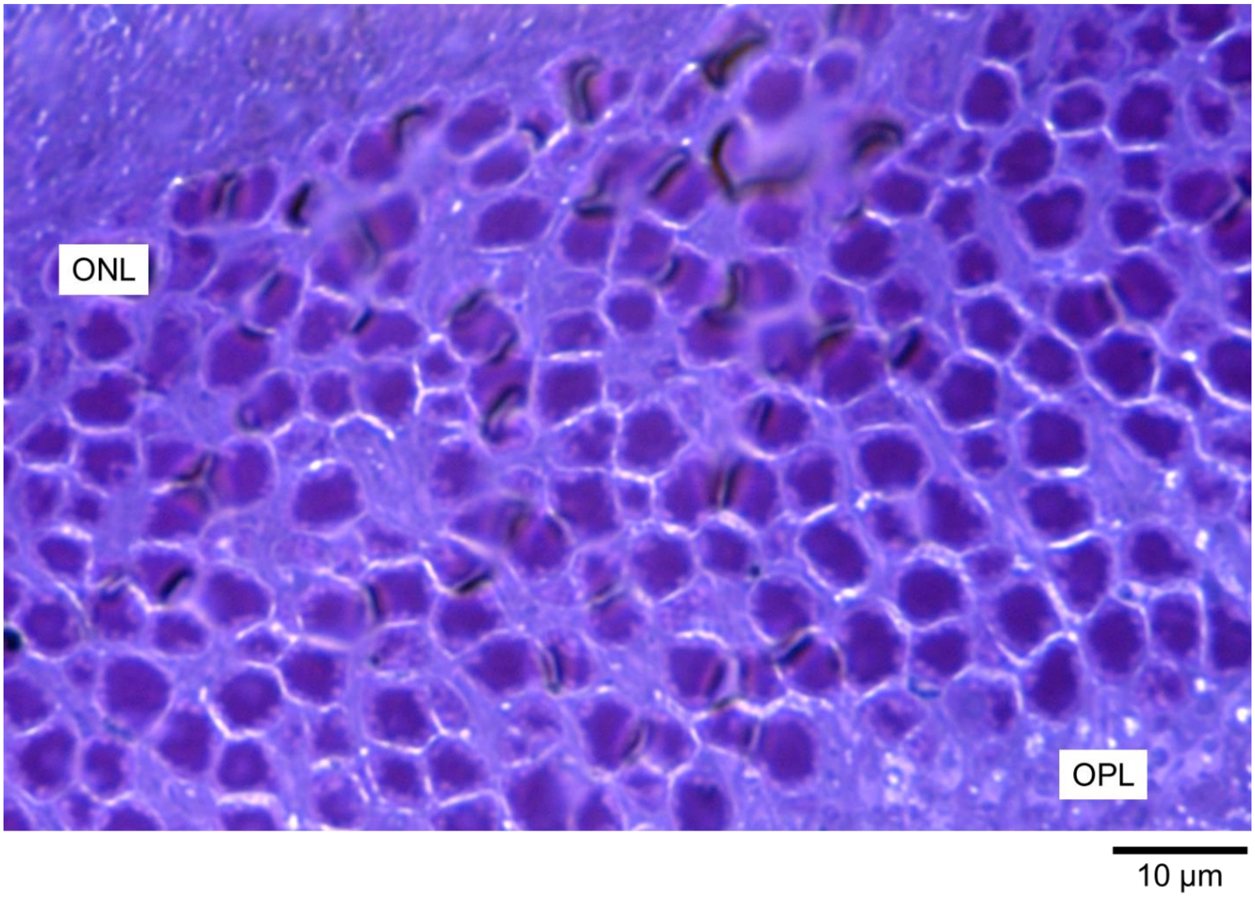

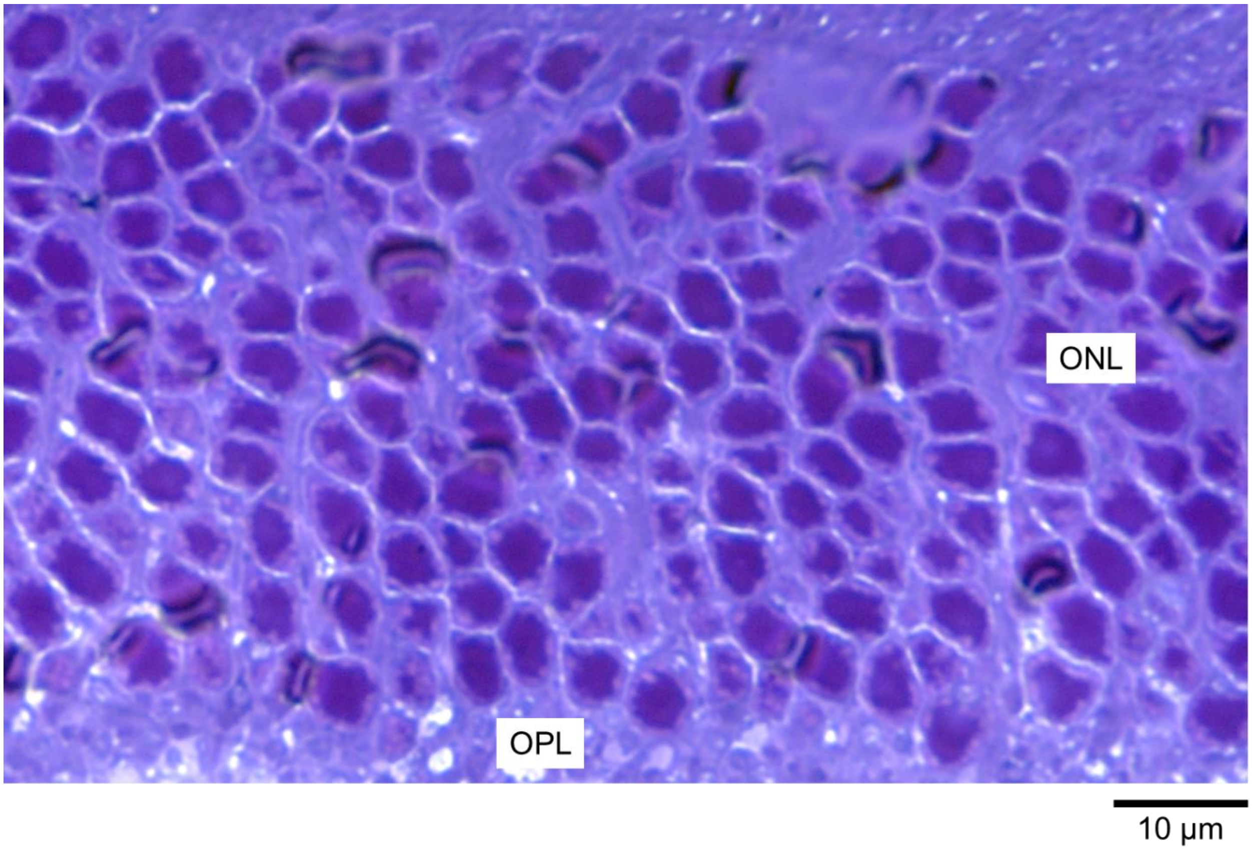
ONL
OPL
10 µm

60 days INL

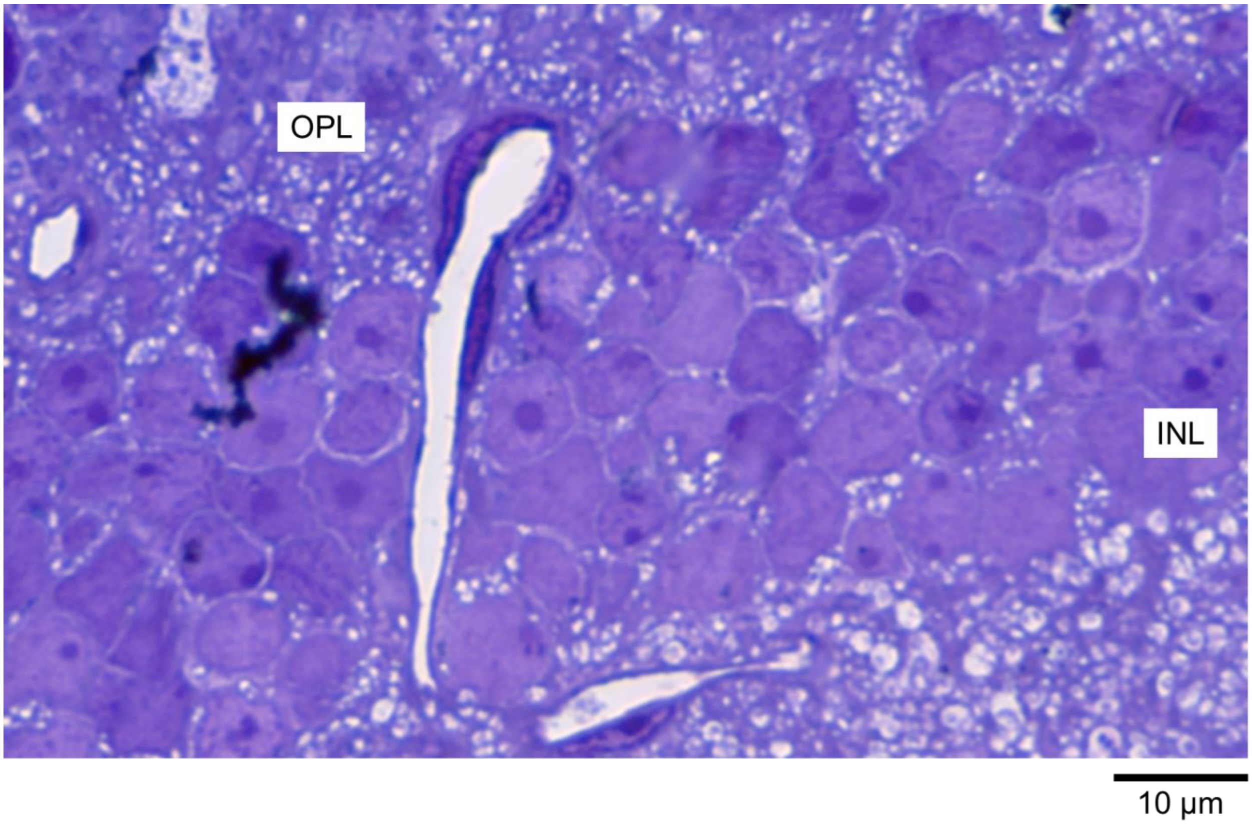

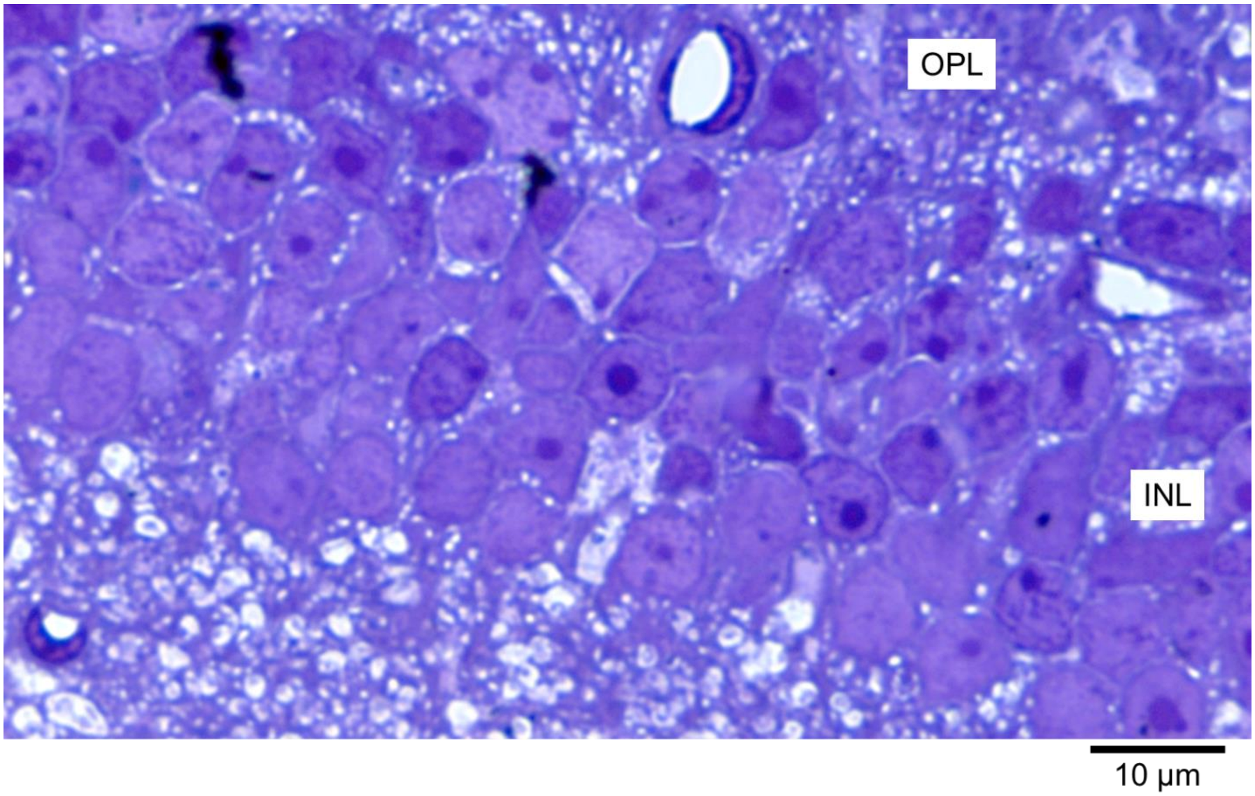

Histograms of Average N Ratios

5 days

5 day ONL Nucleus

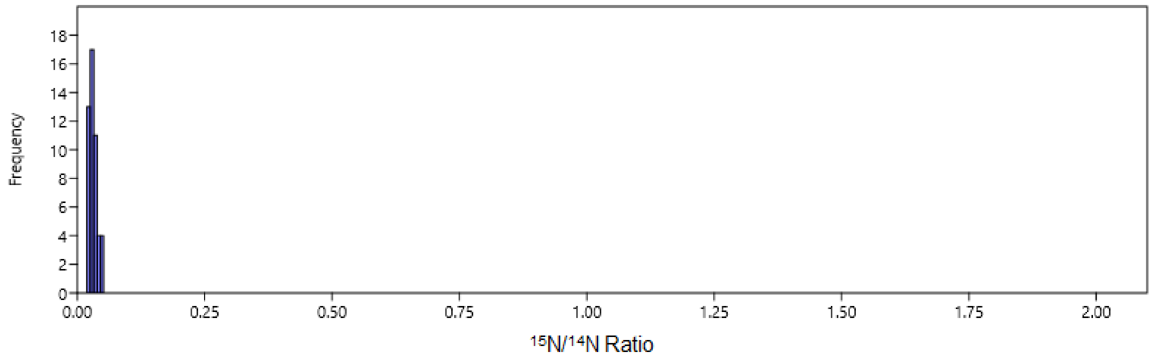

5 day ONL Cytosol

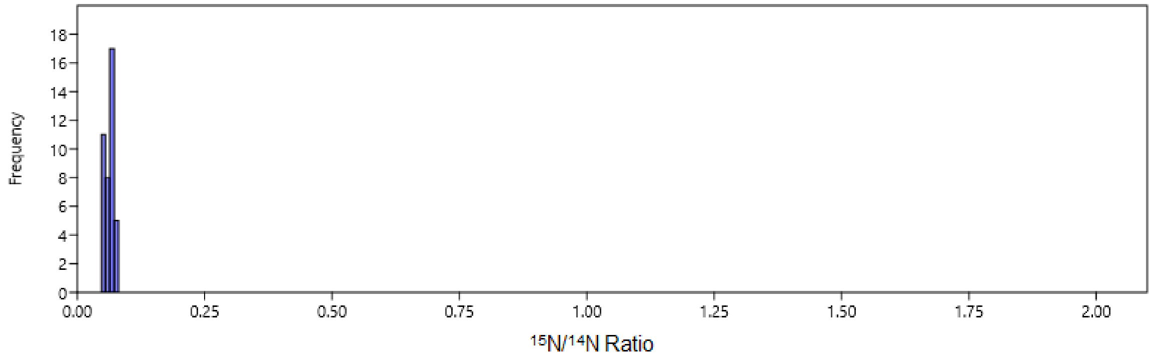

5 day INL Nucleus

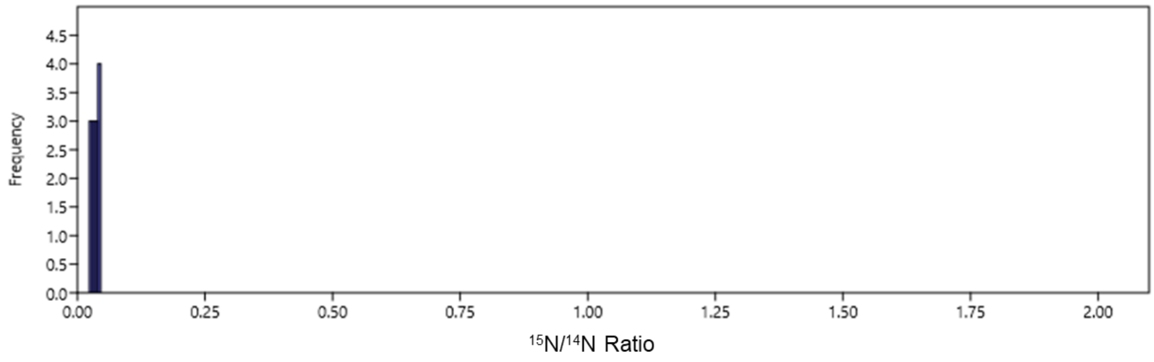

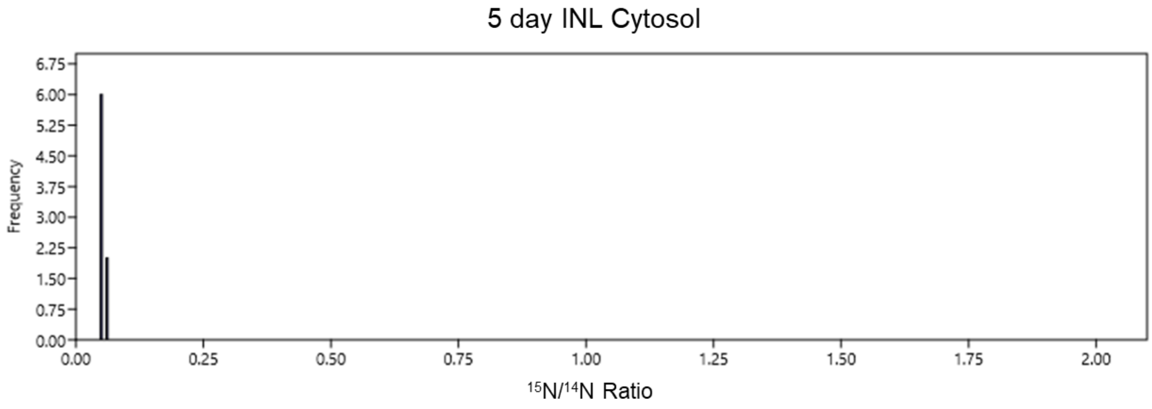
5 day INL Cytosol
Frequency
6.75
6.00
5.25
4.50
3.75
3.00
2.25
1.50
0.75
0.00
0.00
0.25
0.50
0.75
1.00
1.25
1.50
1.75
2.00
15N/14N Ratio

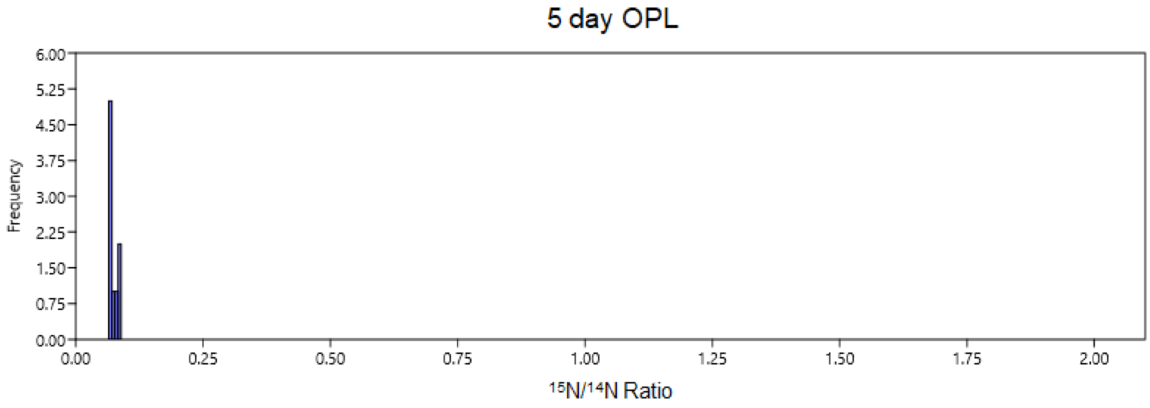
5 day OPL
Frequency
6.00
5.25
4.50
3.75
3.00
2.25
1.50
0.75
0.00
0.00
0.25
0.50
0.75
1.00
1.25
1.50
1.75
2.00
15N/14N Ratio

14 days

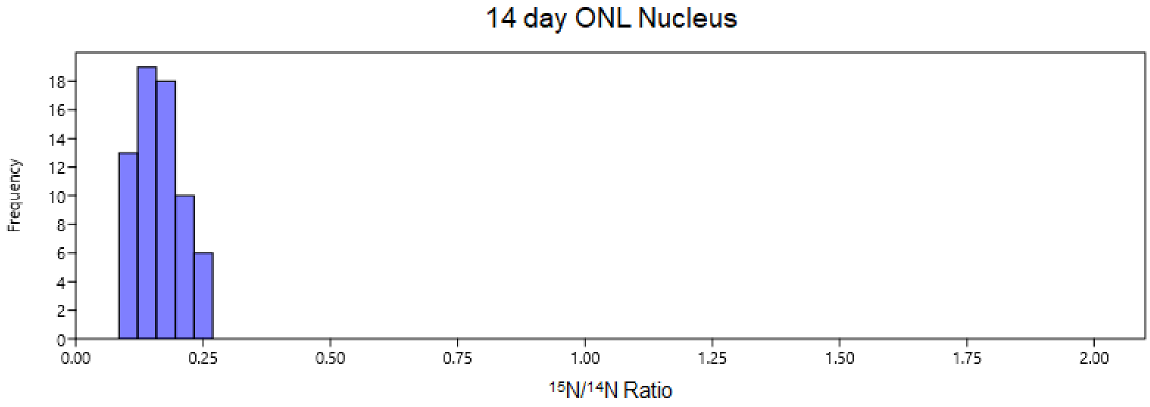
14 day ONL Nucleus
Frequency
18
16
14
12
10
8
6
4
2
0
0.00
0.25
0.50
0.75
1.00
1.25
1.50
1.75
2.00
15N/14N Ratio

14 day ONL Cytosol

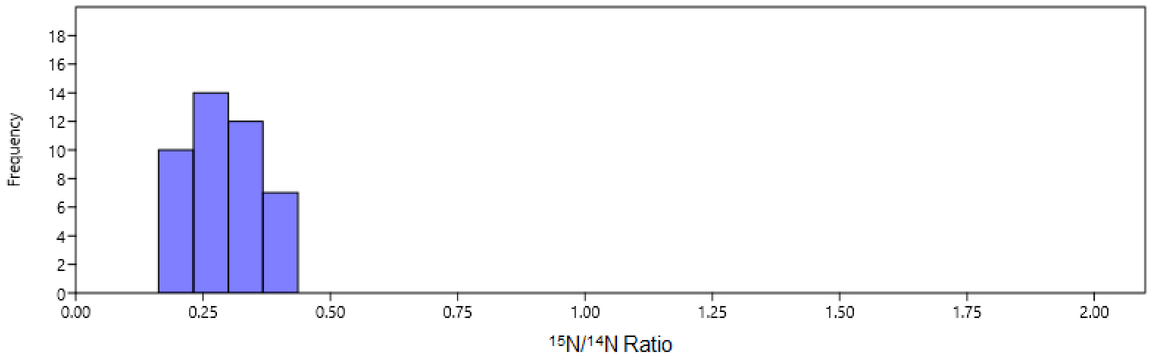

14 day INL Nucleus

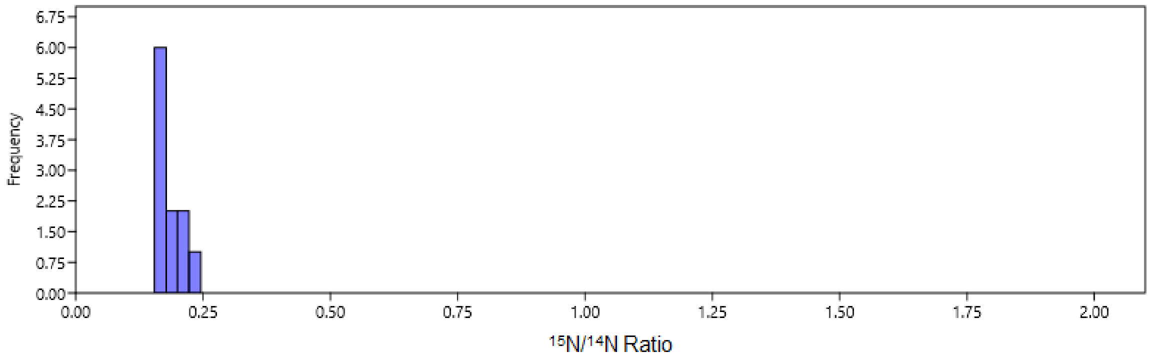

14 day INL Cytosol

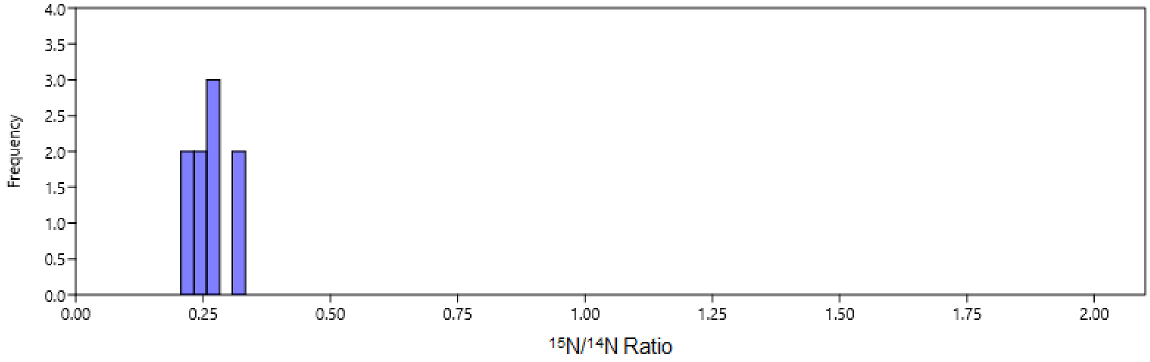

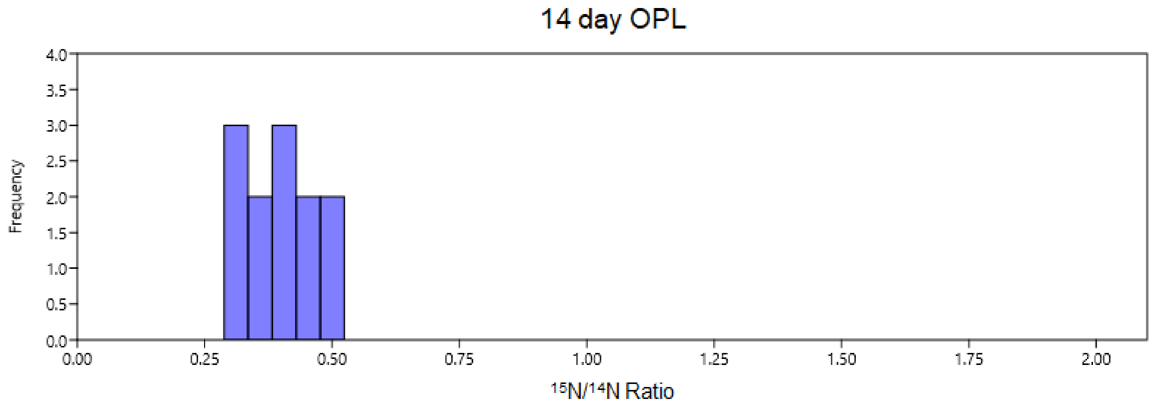
14 day OPL
Frequency
4.0
3.5
3.0
2.5
2.0
1.5
1.0
0.5
0.0
0.00
0.25
0.50
0.75
1.00
1.25
1.50
1.75
2.00
$^{15}N/^{14}N$ Ratio

21 days

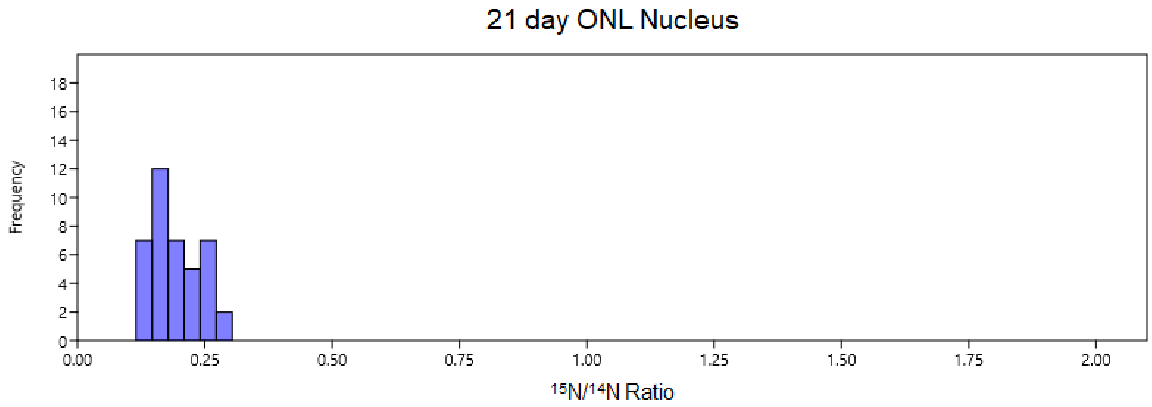
21 day ONL Nucleus
Frequency
18
16
14
12
10
8
6
4
2
0
0.00
0.25
0.50
0.75
1.00
1.25
1.50
1.75
2.00
$^{15}N/^{14}N$ Ratio

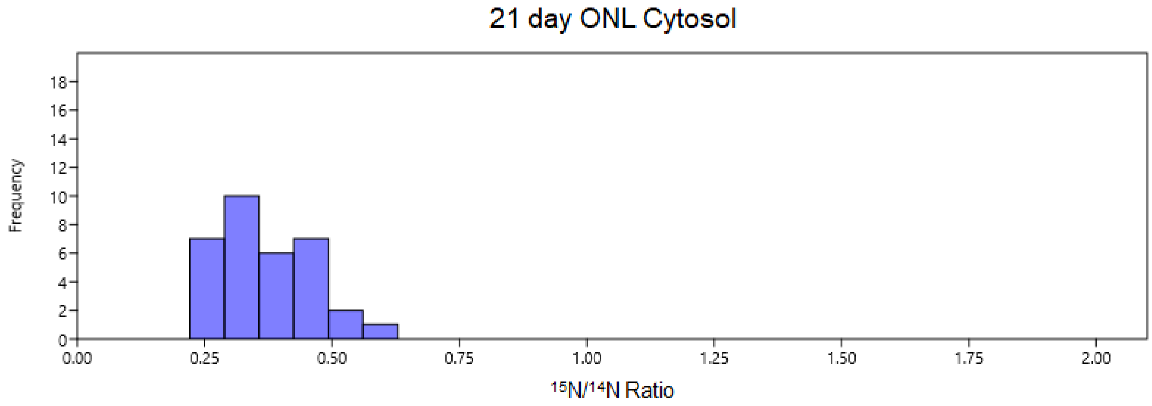
21 day ONL Cytosol
Frequency
18
16
14
12
10
8
6
4
2
0
0.00
0.25
0.50
0.75
1.00
1.25
1.50
1.75
2.00
$^{15}N/^{14}N$ Ratio

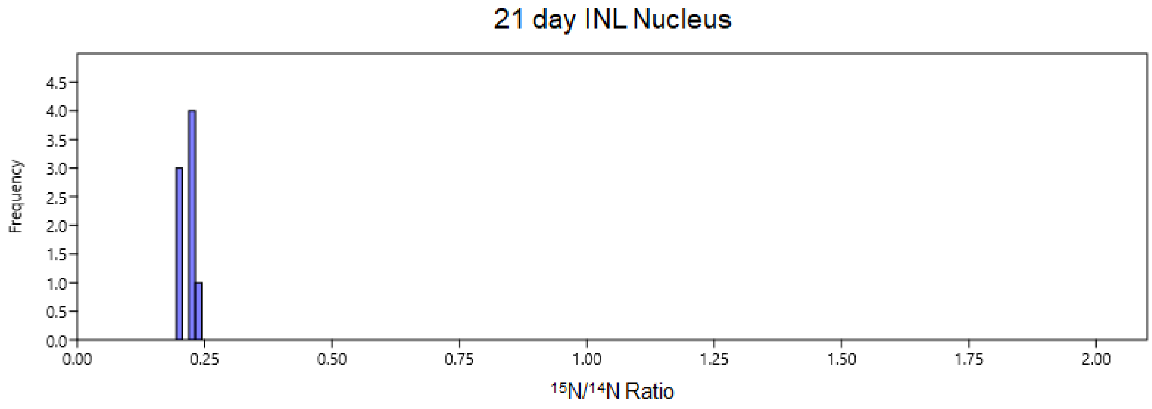
21 day INL Nucleus
Frequency
4.5
4.0
3.5
3.0
2.5
2.0
1.5
1.0
0.5
0.0
0.00
0.25
0.50
0.75
1.00
1.25
1.50
1.75
2.00
15N/14N Ratio

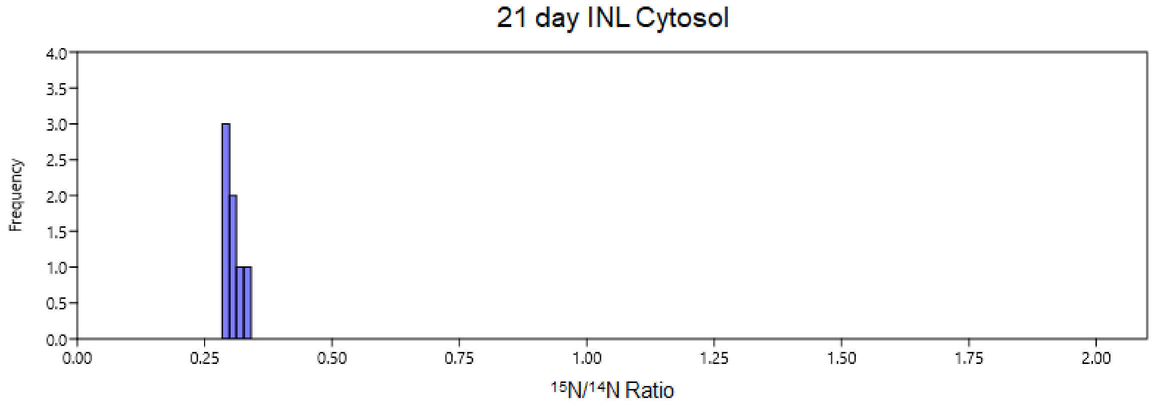
21 day INL Cytosol
Frequency
4.0
3.5
3.0
2.5
2.0
1.5
1.0
0.5
0.0
0.00
0.25
0.50
0.75
1.00
1.25
1.50
1.75
2.00
15N/14N Ratio

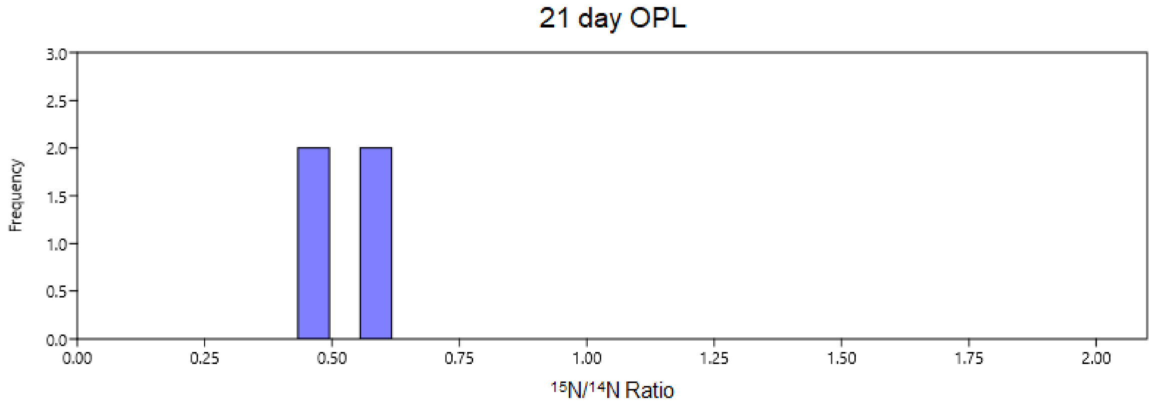
21 day OPL
Frequency
3.0
2.5
2.0
1.5
1.0
0.5
0.0
0.00
0.25
0.50
0.75
1.00
1.25
1.50
1.75
2.00
15N/14N Ratio

60 days

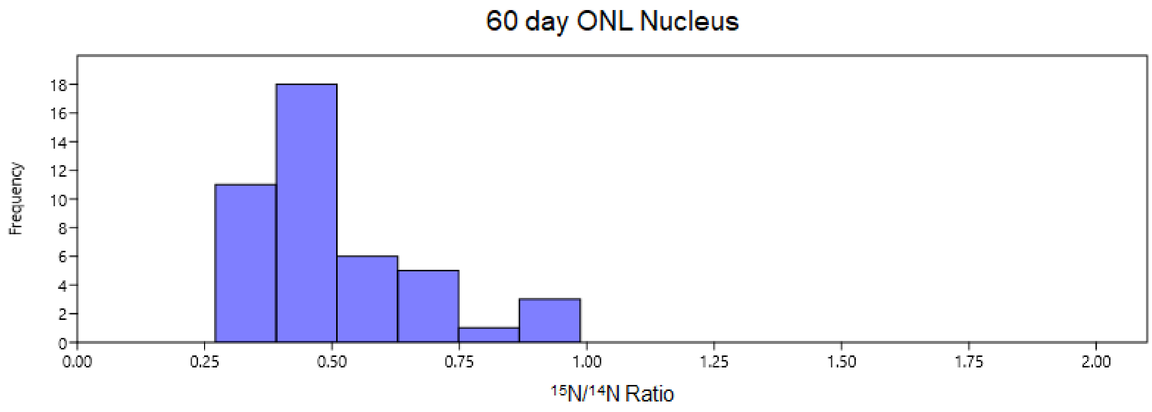
60 day ONL Nucleus
Frequency
18
16
14
12
10
8
6
4
2
0
0.00
0.25
0.50
0.75
1.00
1.25
1.50
1.75
2.00
15N/14N Ratio

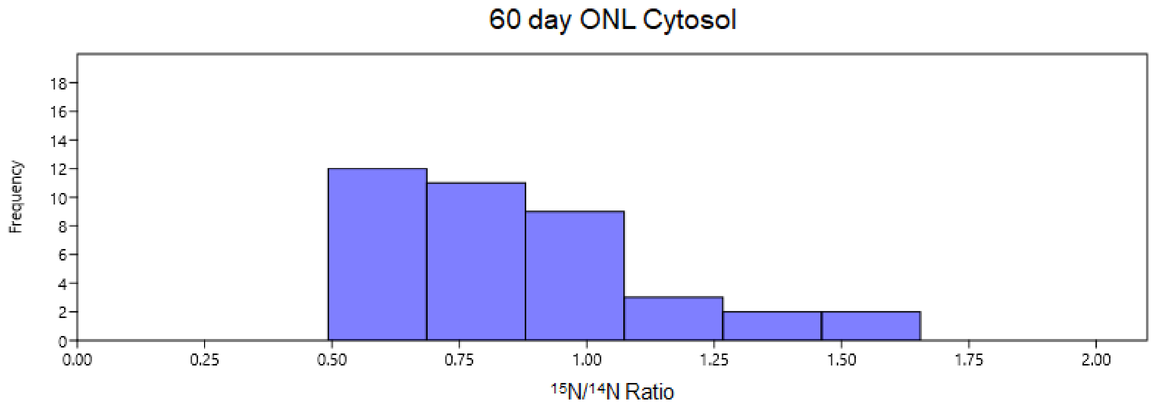
60 day ONL Cytosol
Frequency
18
16
14
12
10
8
6
4
2
0
0.00
0.25
0.50
0.75
1.00
1.25
1.50
1.75
2.00
15N/14N Ratio

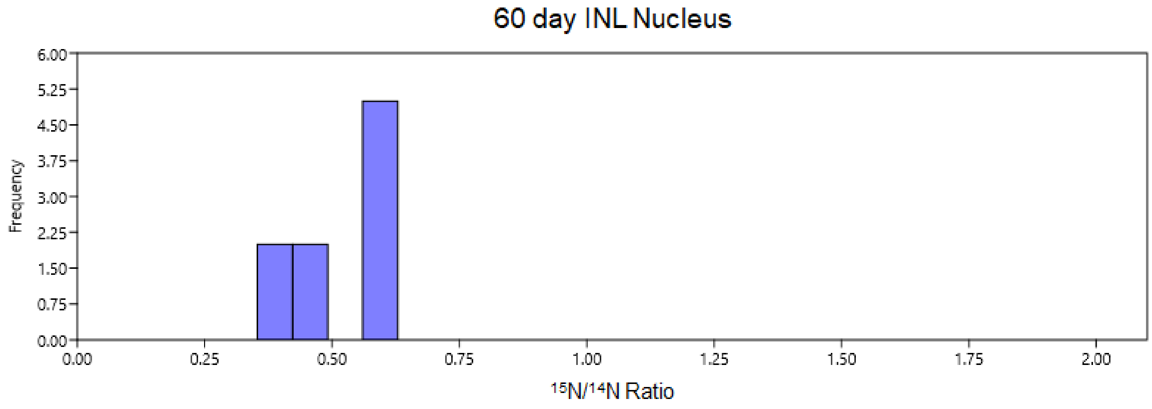
60 day INL Nucleus
Frequency
6.00
5.25
4.50
3.75
3.00
2.25
1.50
0.75
0.00
0.00
0.25
0.50
0.75
1.00
1.25
1.50
1.75
2.00
15N/14N Ratio

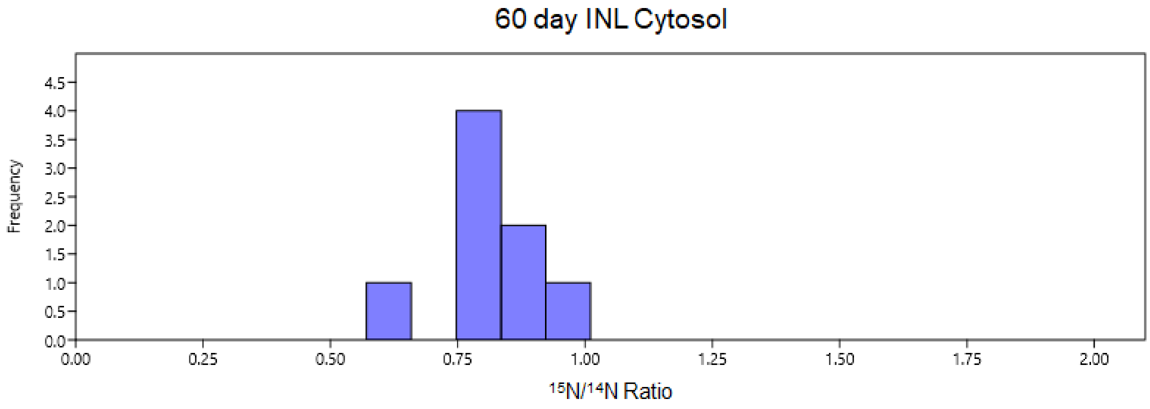

60 day INL Cytosol
Frequency
15N/14N Ratio

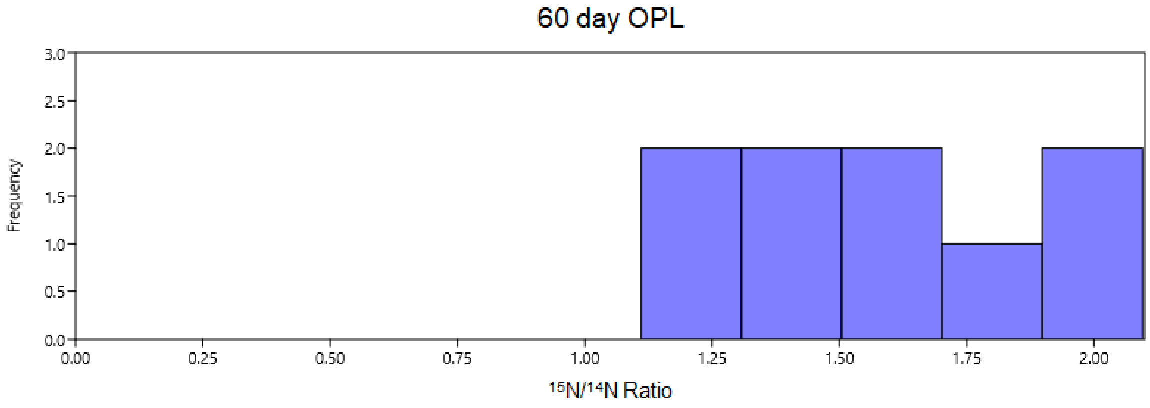

60 day OPL
Frequency
15N/14N Ratio

P56 Mice

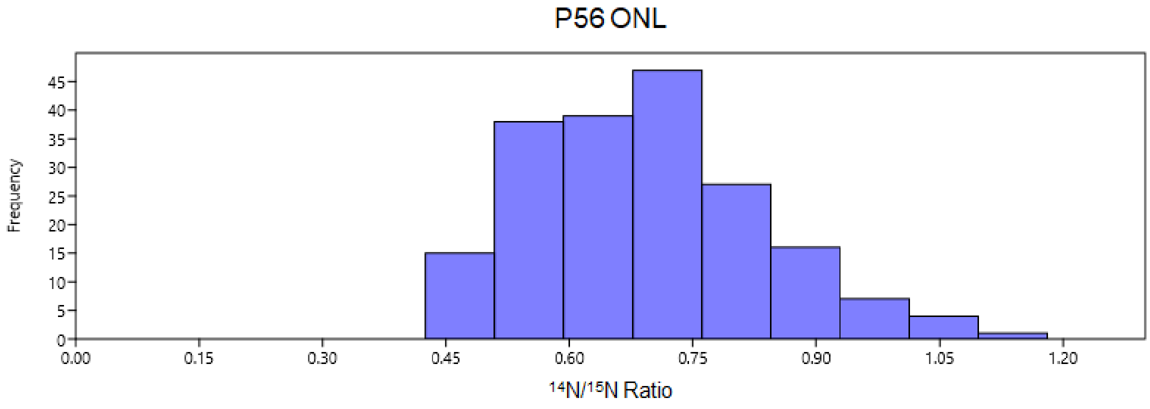

P56 ONL
Frequency
14N/15N Ratio

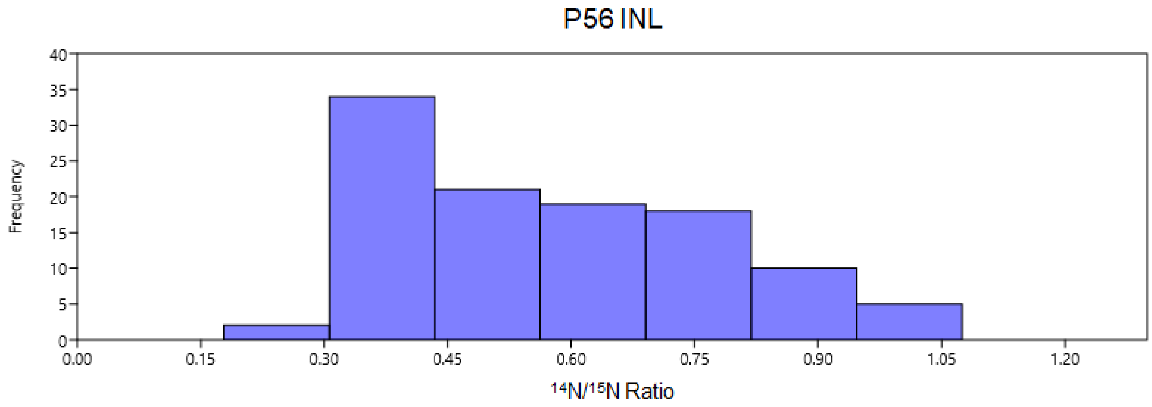
P56 INL
40
35
30
25
20
15
10
5
0
Frequency
0.00
0.15
0.30
0.45
0.60
0.75
0.90
1.05
1.20
$^{14}N/^{15}N$ Ratio

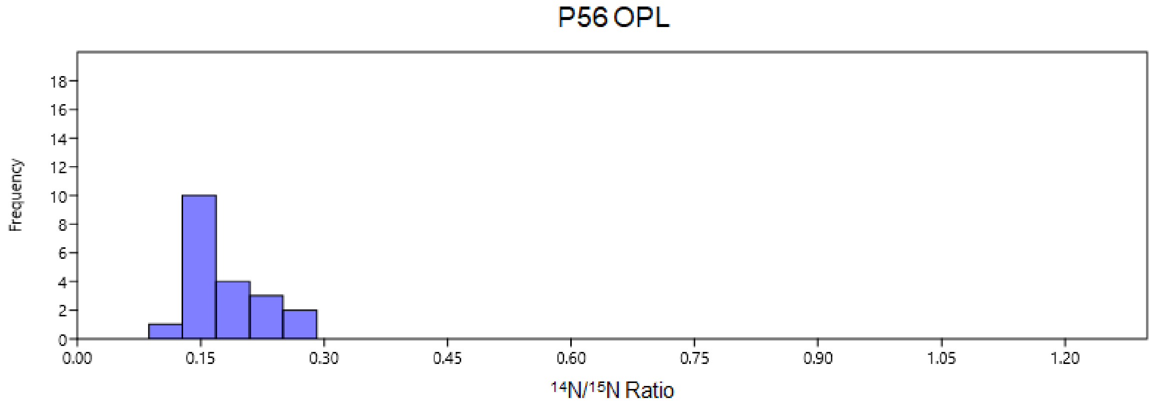
P56 OPL
18
16
14
12
10
8
6
4
2
0
Frequency
0.00
0.15
0.30
0.45
0.60
0.75
0.90
1.05
1.20
$^{14}N/^{15}N$ Ratio
